# The two-component system ArlRS is essential for wall teichoic acid glycoswitching in *Staphylococcus aureus*

**DOI:** 10.1128/mbio.02668-24

**Published:** 2024-11-29

**Authors:** Marieke M. Kuijk, Emma Tusveld, Esther Lehmann, Rob van Dalen, Iñigo Lasa, Hanne Ingmer, Yvonne Pannekoek, Nina M. van Sorge

**Affiliations:** 1 Department of Medical Microbiology and Infection Prevention, Amsterdam University Medical Center, University of Amsterdam, Amsterdam, the Netherlands; 2 Department of Veterinary and Animal Sciences, University of Copenhagen, Copenhagen, Denmark; 3 Laboratory of Microbial Pathogenesis, Navarrabiomed, Universidad Pública de Navarra, Complejo Hospitalario de Navarra, IdiSNA, Pamplona, Navarra, Spain; 4 Netherlands Reference Laboratory for Bacterial Meningitis, Amsterdam University Medical Center location AMC, Amsterdam, the Netherlands; St. Jude Children's Research Hospital, Memphis, Tennessee, USA

**Keywords:** *Staphylococcus aureus*, wall teichoic acid, glycosylation, host-pathogen interactions, virulence regulation, two-component regulatory systems

## Abstract

*Staphylococcus aureus* is among the leading causes of hospital-acquired infections. Critical to *S. aureus* biology and pathogenesis are the cell wall-anchored glycopolymers wall teichoic acids (WTA). Approximately one-third of *S. aureus* isolates decorates WTA with a mixture of α1,4- and β1,4-*N*-acetylglucosamine (GlcNAc), which requires the dedicated glycosyltransferases TarM and TarS, respectively. Environmental conditions, such as high salt concentrations, affect the abundance and ratio of α1,4- and β1,4-GlcNAc WTA decorations, thereby impacting biological properties such as antibody binding and phage infection. To identify regulatory mechanisms underlying WTA glycoswitching, we screened 1,920 *S*. *aureus* mutants (Nebraska Transposon Mutant Library) by immunoblotting for differential expression of WTA-linked α1,4- or β1,4-GlcNAc using specific monoclonal antibody Fab fragments. Three two-component systems (TCS), GraRS, ArlRS, and AgrCA, were among the 230 potential hits. Using isogenic TCS mutants, we demonstrated that ArlRS is essential for WTA β1,4-GlcNAc decoration. ArlRS repressed *tarM* expression through the transcriptional regulator MgrA. In bacteria lacking *arlRS*, the increased expression of *tarM* correlated with the absence of WTA β1,4-GlcNAc, likely by outcompeting TarS enzymatic activity. ArlRS was responsive to Mg^2+^, but not Na^+^, revealing its role in the previously reported salt-induced WTA glycoswitch from α1,4-GlcNAc to β1,4-GlcNAc. Importantly, ArlRS-mediated regulation of WTA glycosylation affected *S. aureus* interaction with the innate receptor langerin and lysis by β1,4-GlcNAc-dependent phages. Since WTA represents a promising target for future immune-based treatments and vaccines, our findings provide important insight to align strategies targeting *S. aureus* WTA glycosylation patterns during infection.

**IMPORTANCE:**

*Staphylococcus aureus* is a common colonizer but can also cause severe infections in humans. The development of antibiotic resistance complicates the treatment of *S. aureus* infections, increasing the need for antibiotic alternatives such as vaccines and therapies with bacterial viruses also known as phages. Wall teichoic acids (WTA) are abundant glycosylated structures of the *S. aureus* cell wall that have gained attention as a promising target for new treatments. Importantly, WTA glycosylation patterns show variation depending on environmental conditions, thereby impacting phage binding and interaction with host factors, such as antibodies and innate pattern-recognition receptors. Here, we show that the two-component system ArlRS is involved in the regulation of WTA glycosylation by responding to environmental changes in Mg^2+^ concentration. These findings may support the design of new treatment strategies that target WTA glycosylation patterns of *S. aureus* during infection.

## INTRODUCTION

Antimicrobial resistance is a global health crisis, which is estimated to cause an increasing number of deaths worldwide in the upcoming years (1). Infections caused by the Gram-positive pathogen *Staphylococcus aureus* are associated with high morbidity and mortality rates, and methicillin-resistant *S. aureus* specifically accounted for more than 100,000 deaths globally in 2019 (1). Consequently, *S. aureus* is one of six pathogens that significantly contributes to the number of multidrug-resistant deaths and is classified by the WHO as a high-priority pathogen (2). Novel treatments to combat these infections are therefore urgently needed.

Wall teichoic acids (WTA) are critical glycopolymers for *S. aureus* cell wall architecture, physiology, and host interaction. WTA molecules are covalently anchored to peptidoglycan and are composed of polyribitol-phosphate decorated with *N*-acetylglucosamine (GlcNAc) and D-alanine. WTA glycosylation plays an important role in a wide range of processes, including β-lactam resistance (3), phage infectivity (4, 5), and host interactions such as nasal colonization (6, 7), detection by WTA-specific antibodies (8–10), and the langerin receptor on skin Langerhans cells (11). The GlcNAc residues can be linked in several distinct orientations, resulting in structural heterogeneity of WTA. Nearly all *S. aureus* isolates glycosylate WTA with β1,4-GlcNAc through the activity of the specific housekeeping glycosyltransferase TarS (3, 12). However, approximately one-third of *S. aureus* isolates can co-decorate WTA with α1,4-GlcNAc through the activity of the glycosyltransferase TarM (12, 13). The *tarS* gene is part of the core genome and is co-localized with several other WTA biosynthesis genes, whereas *tarM* is located elsewhere in the genome (3, 14). Importantly, several of the WTA-mediated processes, for example, β-lactam resistance, langerin binding, and infection by specific phages, are dependent on WTA β1,4-GlcNAc modification and may even be blocked by co-decoration with α1,4-GlcNAc (15–17). The β1,4-GlcNAc moiety is also a dominant target for human antibodies and is therefore an interesting target for immune-mediated treatments and vaccines (8, 10, 18). Clearly, these findings illustrate how changes in WTA glycosylation affect and shape host-pathogen interactions.

*S. aureus* can rapidly adapt its surface properties to different environmental conditions encountered during its commensal and pathogenic lifestyles, which is a major challenge for treating infections (19, 20). One of these adaptations relates to different glycosylation patterns of the cell wall that may favor bacterial survival in specific niches. Indeed, *S. aureus* can shift the abundance of α1,4-GlcNAc and β1,4-GlcNAc WTA glycosylation depending on the environmental conditions. For example, bacteria grown *in vitro* under rich growth conditions primarily decorate WTA with α1,4-GlcNAc, whereas *S. aureus* isolated from organs after murine infection or after culture in high salt conditions express predominantly β1,4-GlcNAc WTA (8, 21). Culture density also affects α1,4-/β1,4-GlcNAc WTA glycosylation ratios through quorum sensing, as auto-inducing peptides have been observed to reduce *tarM* expression in stationary cultures, resulting in increased phage-mediated lysis (22). Although these examples illustrate the ability of *S. aureus* to switch between α1,4- or β1,4-GlcNAc-dominated WTA glycosylation profiles, the underlying molecular mechanisms and regulatory pathways are currently unknown.

Key to the virulence of *S. aureus* as a pathogen is its ability to quickly adapt its gene expression profile (23, 24) through a network of transcriptional regulators (SarA, Rot, MgrA, among others), alternative sigma factors (SigB and SigH) (23) and two-component systems (TCS) (25). *S. aureus* encodes 16 TCS, of which only WalRK is essential (25, 26). TCS recognize specific external triggers by a sensing histidine kinase, leading to transcriptional regulation through the phosphorylated response regulator (27). Intriguingly, no major growth defects were observed in a *S. aureus* mutant lacking all 15 non-essential TCS under standard laboratory conditions (25). However, many TCS, such as AgrCA, SaeRS, SrrAB, and ArlRS, have been shown to increase bacterial virulence or survival in certain *in vivo* conditions (23, 27). With regard to WTA glycosylation, only AgrCA has been experimentally implicated in the transcriptional regulation of *tarM* (22). Also, GraRS has also been implicated in the regulation of *tarM* (28), although this has not been functionally verified at the level of WTA glycosylation.

The TCS ArlRS has also been linked to WTA, but as a repressor of the *dlt* operon encoding the machinery for WTA D-alanylation (29). In addition, ArlRS is important in regulating host interaction processes such as adhesion and damage to endothelial cells (30, 31), clumping with fibrinogen (31–33), and expression of several immune evasion factors that block neutrophil killing (34). Although the exact triggers to activate ArlRS in *S. aureus* are not yet known, ArlRS has been implicated in the homeostasis of the essential divalent cations Mn^2+^ and Mg^2+^ (29, 35, 36).

The regulatory mechanisms that control WTA glycoswitching in response to varying environmental conditions remain largely unknown. This study aimed to identify key regulators and unravel the molecular mechanisms involved in the differential expression of α1,4- and β1,4-GlcNAc phenotypes. We demonstrated that ArlRS is essential for β1,4-GlcNAc decoration of WTA, which impacted phage infection and langerin interaction. In addition, ArlRS was required for the Mg^2+^-induced glycoswitch from α1,4- to β1,4-GlcNAc WTA glycosylation *via* the transcription factor MgrA. Given that WTA represents a promising target for future immune-based treatments and vaccines, our findings are of significance when designing new strategies that align with the WTA glycosylation patterns of *S. aureus* during infection.

## RESULTS

### Environmental conditions affect *tarM*, but not *tarS* transcription levels

**Fig 1:**
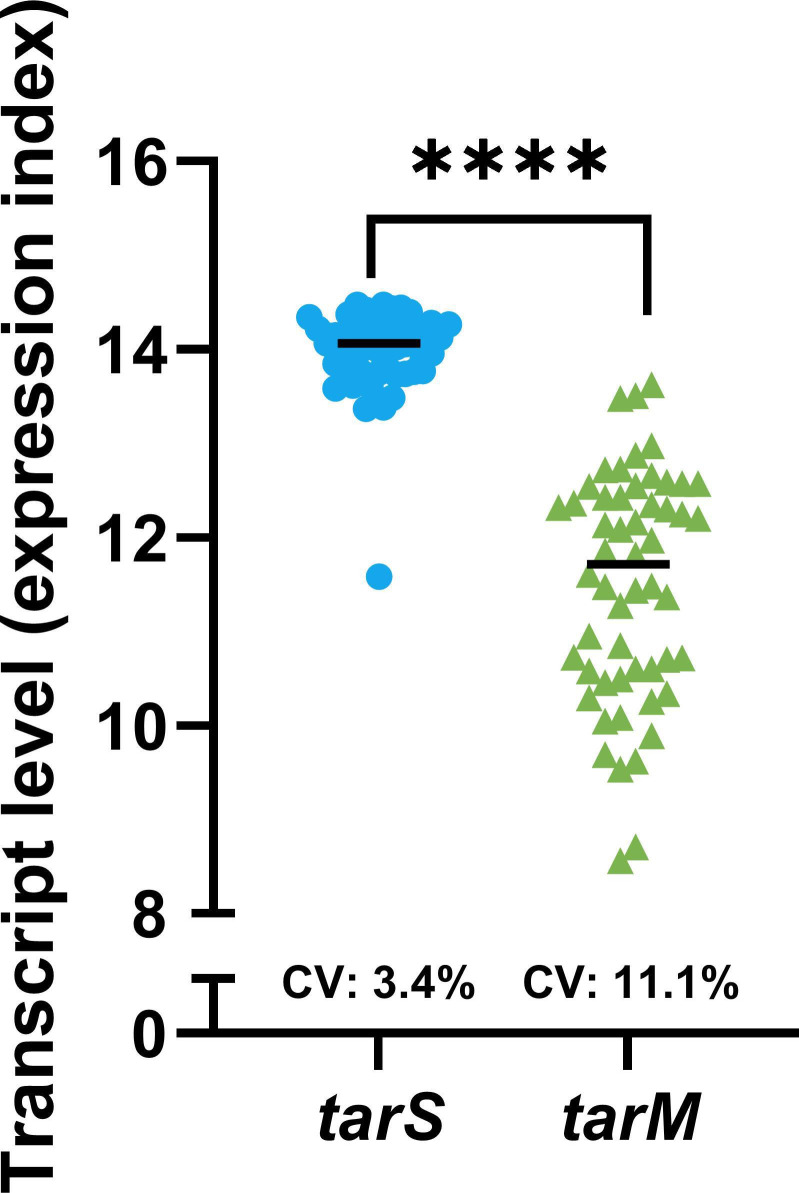
*tarM* expression is dynamically regulated. Transcript levels of *tarS* (SAOUHSC_00228) and *tarM* (SAOUHSC_00973) of *S. aureus* HG001 in 44 different *in vitro* and *in vivo*-mimicking conditions. The coefficient of variation (CV) of both data sets is shown. Data are extracted from Mäder *et al*. (24, 37). The variance of the two genes was statistically tested using an F test. ****P < 0.0001.

*S. aureus* strains carrying both *tarM* and *tarS* can co-decorate WTA with α1,4- and β1,4-GlcNAc, respectively. To understand the molecular mechanisms underlying the modification of the α1,4- and β1,4-GlcNAc ratio in response to environmental conditions (21), we investigated how expression levels of *tarM* or *tarS* are affected in different environments. We therefore re-analyzed a previously published transcriptomics data set of *S. aureus* strain HG001 across 44 different conditions ranging from *in vitro* laboratory culture to *in vivo*-mimicking host environments (24). From this data set, we extracted transcription data for the *tarM* and *tarS* genes (37) and compared the variance of the expression levels of the two genes across these conditions. Expression of *tarM* fluctuated substantially, and this variance significantly differed from the more stable *tarS* expression in these 44 experimental conditions (Fig. 1). These results suggest that shifts in the WTA glycosylation profile are predominantly regulated through *tarM* rather than *tarS*.

### Multiple two-component systems are identified to be involved in WTA glycoswitching

**Fig 2:**
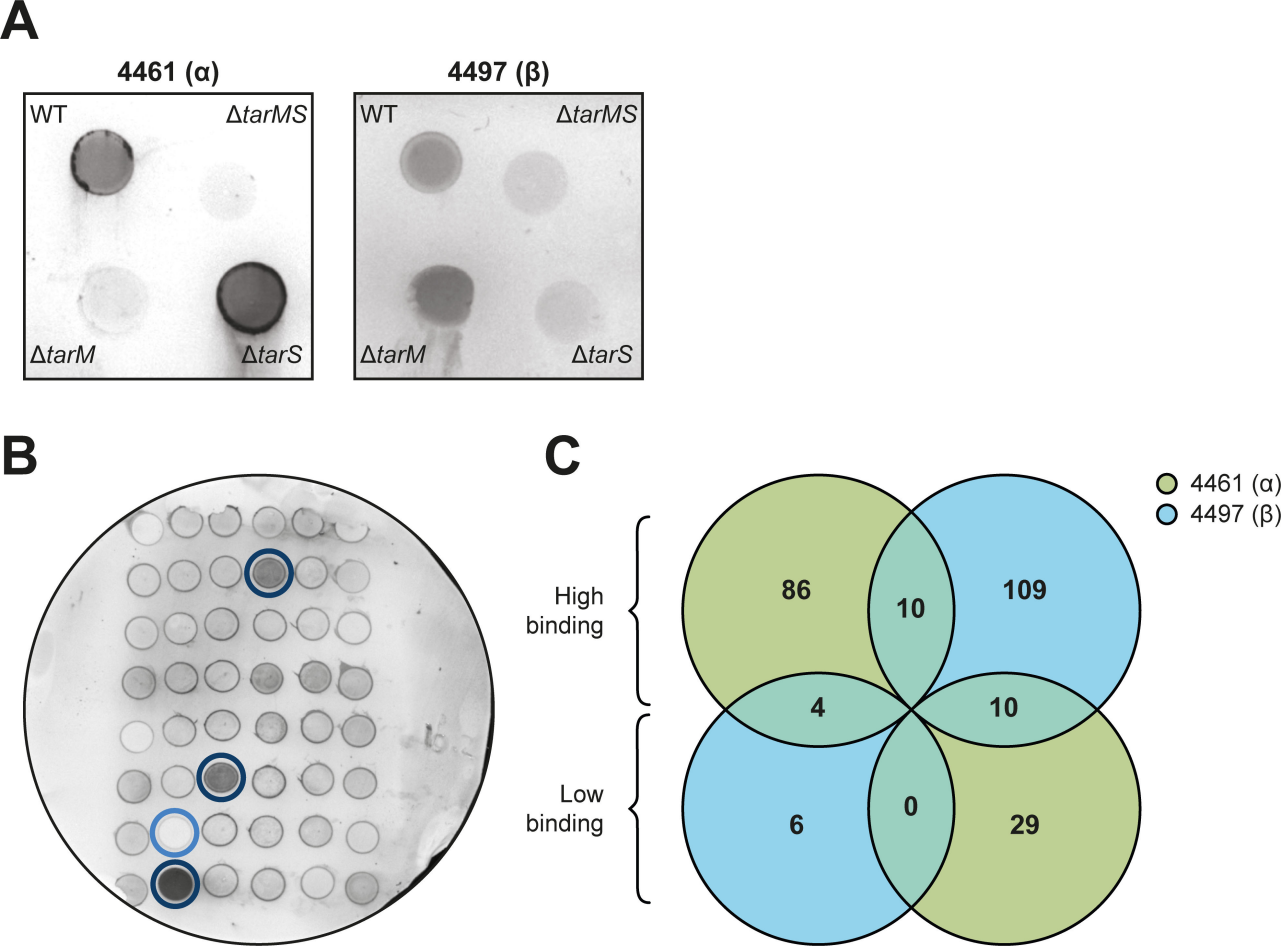
Representative immunoblot and Venn diagram summarizing results from the NTML screen to identify WTA glycoswitch mutants. (**A**) Representative immunoblot of JE2 WT and deletion mutants Δ*tarMS*, Δ*tarM* and Δ*tarS* in JE2 background stained with 4461 or 4497 (**B**) Representative image of an immunoblot containing 48 NTML mutants screened for binding of 4461 (α1,4-GlcNAc). Three transposon mutants show comparatively high binding (encircled in dark blue) and one transposon mutant shows low binding (encircled in light blue). (**C**) Venn diagram with a number of NTML mutants identified with either high or low levels of α1,4- (in blue) and β1,4-glycosylation (in green). The number of overlapping mutants is also depicted.

Given the dynamic regulation of *tarM*, we next aimed to identify the genes that may be involved in this process. To this end, we screened the Nebraska Transposon Mutant Library (NTML), containing 1,920 arrayed mutants, for differential α1,4- and β1,4-GlcNAc expression (38). Importantly, the NTML parental strain JE2 harbors both *tarM* and *tarS* genes. To facilitate high-throughput screening, we used immunoblotting of individual transposon mutants. A representative control immunoblot containing JE2 wild type (WT) and the markerless deletion mutants Δ*tarMS*, Δ*tarM,* and Δ*tarS* in the same background is visualized in Fig. 2A. We then performed two parallel screens using Fab fragments that specifically detect α1,4- and β1,4-GlcNAc WTA (Fig. 2B) (39).

We identified 230 genes potentially involved in WTA glycosylation (Table S1). Importantly, the transposon mutants *tarM* (SAUSA300_0939) and *tarS* (SAUSA300_0252) were identified as non-binding mutants in their respective 4461 and 4497 screens, thereby acting as positive controls of this unbiased screening method. Among the 230 potential genes, 115 were identified in the α1,4-GlcNAc and 115 in the β1,4-GlcNAc screen (Fig. 2C; Table S1). A higher number of transposon mutants exhibited upregulated WTA glycosylation compared to the number of mutants in which it was downregulated (195 compared to 35; Fig. 2C). Ten mutants showed high binding to both Fabs, whereas 14 genes had a differential effect on α1,4- and β1,4-GlcNAcylation (Fig. 2C). Of interest, we identified six genes of three different TCS (GraRS, ArlRS, and AgrCA) in the 4461 (α1,4-GlcNAc) screen (Table 1). In addition, nine transcriptional regulators were identified across both screens (Table 1).

**TABLE 1:** Identified transposon mutants in two-component systems and regulatory proteins^*a*^

Accession No.	NTML ID	Gene	Description	Fab binding	
				4461 (α)	4497 (β)
Two-component systems					
SAUSA300_0646	NE1756	*graS*	Sensor protein kinase GraS	High	-
SAUSA300_1307	NE1183	*arlS*	Sensor histidine kinase protein	High	-
SAUSA300_1308	NE1684	*arlR*	DNA-binding response regulator	High	-
SAUSA300_1989	NE95	*agrB*	Accessory gene regulator protein B	High	-
SAUSA300_1991	NE873	*agrC*	Accessory gene regulator protein C	High	-
SAUSA300_1992	NE1532	*agrA*	Accessory gene regulator protein A	High	-
Regulators					
SAUSA300_0114	NE165	*sarS*	Staphylococcal accessory regulator	Low	High
SAUSA300_0605	NE1193	*sarA*	Accessory regulator A	High	-
SAUSA300_0647	NE645	*vraF*	ABC transporter, ATP-binding protein	High	-
SAUSA300_1708	NE386	*rot*	Staphylococcal accessory regulator Rot	Low	High
SAUSA300_2022	NE1109	*rpoF*	RNA polymerase sigma factor SigB	High	High
SAUSA300_2025	NE1607	*rsbU*	Sigma-B regulation protein	High	High
SAUSA300_2326	NE1304	-	Transcription regulatory protein	Low	-
Other					
SAUSA300_0023	NE1865	*yycI*	YycI protein (regulation WalRK)	High	-
SAUSA300_2075	NE149	*rho*	Transcription termination factor (regulation SaeRS)	-	High

^*a*^ Glycosylation patterns of NTML mutants were assessed with high, low or average (−) levels of Fab binding of 4461 (α1,4-GlcNAc) or 4497 (β1,4-GlcNAc).

### Two-component system ArlRS is essential for WTA β-GlcNAc expression

**Fig 3:**
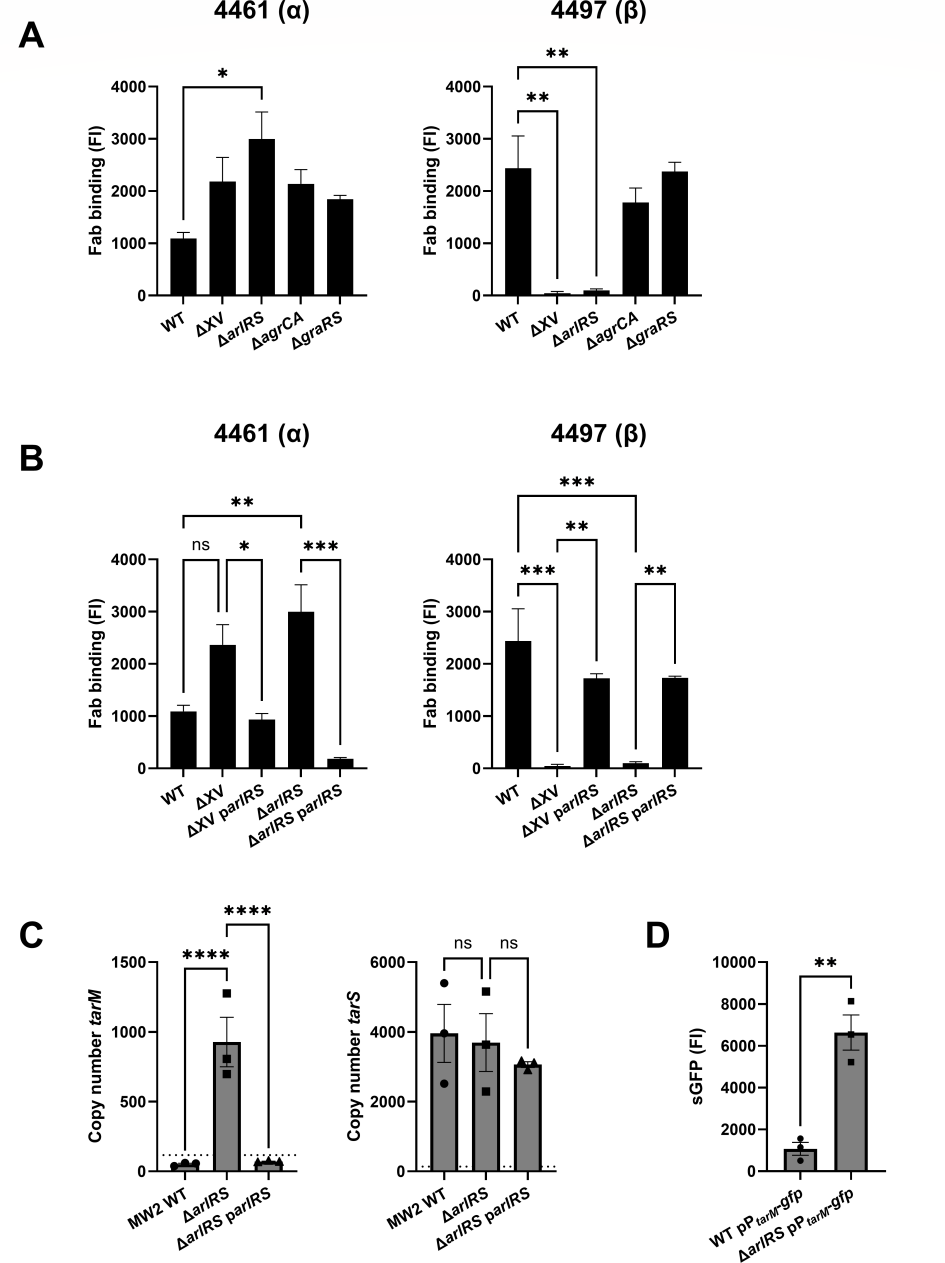
The two-component system ArlRS is essential for WTA β1,4-GlcNAc glycosylation. (**A, B**) Levels of α1,4- and β1,4-GlcNAc, quantified by binding of Fab fragments 4461 (α) and 4497 (β) to (**A**) WT, ΔXV and three single TCS mutants Δ*arlRS*, Δ*agrCA,* and Δ*graRS*, and to (**B**) WT, ΔXV and Δ*arlRS,* and their *arlRS* plasmid-complemented strains. All mutants are in the *S. aureus* MW2 background. Fab binding was analyzed by flow cytometry. (**C**) mRNA copy number of *tarM* and *tarS* as measured with qPCR in WT, Δ*arlRS,* and complemented bacteria. Symbols below the dotted line represent extrapolated values. (**D**) sGFP fluorescence as a measure for promoter activity of *tarM* in WT pP*_tarM_-gfp* or Δ*arlRS* pP*_tarM_-gfp* bacteria as analyzed by flow cytometry and represented as fluorescence intensity (FI). Data are shown as three biological replicates ±SEM. Statistical significance was determined using one-way ANOVA with Bonferroni statistical hypothesis testing to correct for multiple comparisons (**A, B, C**) and Student’s t test (**D**). For panel (**A**), the mean of each column was compared to WT marking only significant comparisons. *P < 0.05, **P < 0.01, ***P < 0.001, ****P < 0.0001, ns = not significant.

Based on the identification of three different TCS, we next aimed to confirm the role of these TCS in regulating WTA glycosylation. We first analyzed 4461 and 4497 Fab binding to a markerless deletion mutant lacking all 15 non-essential TCS (ΔXV) using flow cytometry (25), and observed that this mutant completely lacked WTA β1,4-GlcNAc glycosylation (Fig. 3A). Further analysis of three isogenic TCS mutants, Δ*arlRS,* Δ*agrCA, and* Δ*graRS*, showed that the WTA glycosylation phenotype of the Δ*arlRS* mutant was similar to the ΔXV mutant with regard to β1,4-glycosylation, but additionally showed significantly increased α1,4-GlcNAc levels (Fig. 3A). By contrast, deletion of *graRS* and *agrCA* did not significantly alter α1,4- or β1,4-GlcNAc WTA glycosylation levels (Fig. 3A). The β1,4-GlcNAc-deficient phenotype of ΔXV and Δ*arlRS* was completely rescued through *arlRS* plasmid complementation (Fig. 3B), demonstrating that *arlRS* is essential for the expression of WTA β1,4-GlcNAc expression in our assay conditions. As the growth phase of bacteria can affect glycosylation (22), we compared the growth characteristics of WT bacteria, Δ*arlRS* and Δ*arlRS* p*arlRS* mutants (Fig. S1A and C). No growth deficits were seen in either the growth curves or the CFU per mL overnight culture (Fig. S1). This indicates that the differences in WTA glycosylation are due to the ArlRS TCS rather than culture density changes.

To investigate whether ArlRS regulated the expression of *tarM* or *tarS*, we quantified the absolute copy numbers of *tarM* and *tarS* by qPCR. The Δ*arlRS* mutant showed a substantial increase in *tarM* copy number compared to WT, which was reversed by complementing the mutant with plasmid-expressed *arlRS*. By contrast, no changes were observed between the *tarS* copy numbers of WT, Δ*arlRS,* and the complemented mutant (Fig. 3C). To assess whether increased *tarM* copy numbers correlated with increased promoter activity, we used an sGFP-reporter system in which the *tarM* promoter region was fused to *gfp* in plasmid pCM29 (40). This plasmid was transformed into WT and Δ*arlRS* to create WT pP*_tarM_-gfp* and Δ*arlRS* pP*_tarM_-gfp*. Δ*arlRS* showed a significantly increased *tarM* promoter activity compared to WT (Fig. 3D). These data indicate that ArlRS acts as a repressor of *tarM* expression, promoting WTA β1,4-GlcNAc decoration.

### High Mg^2+^ concentrations drive WTA glycoswitching

**Fig 4:**
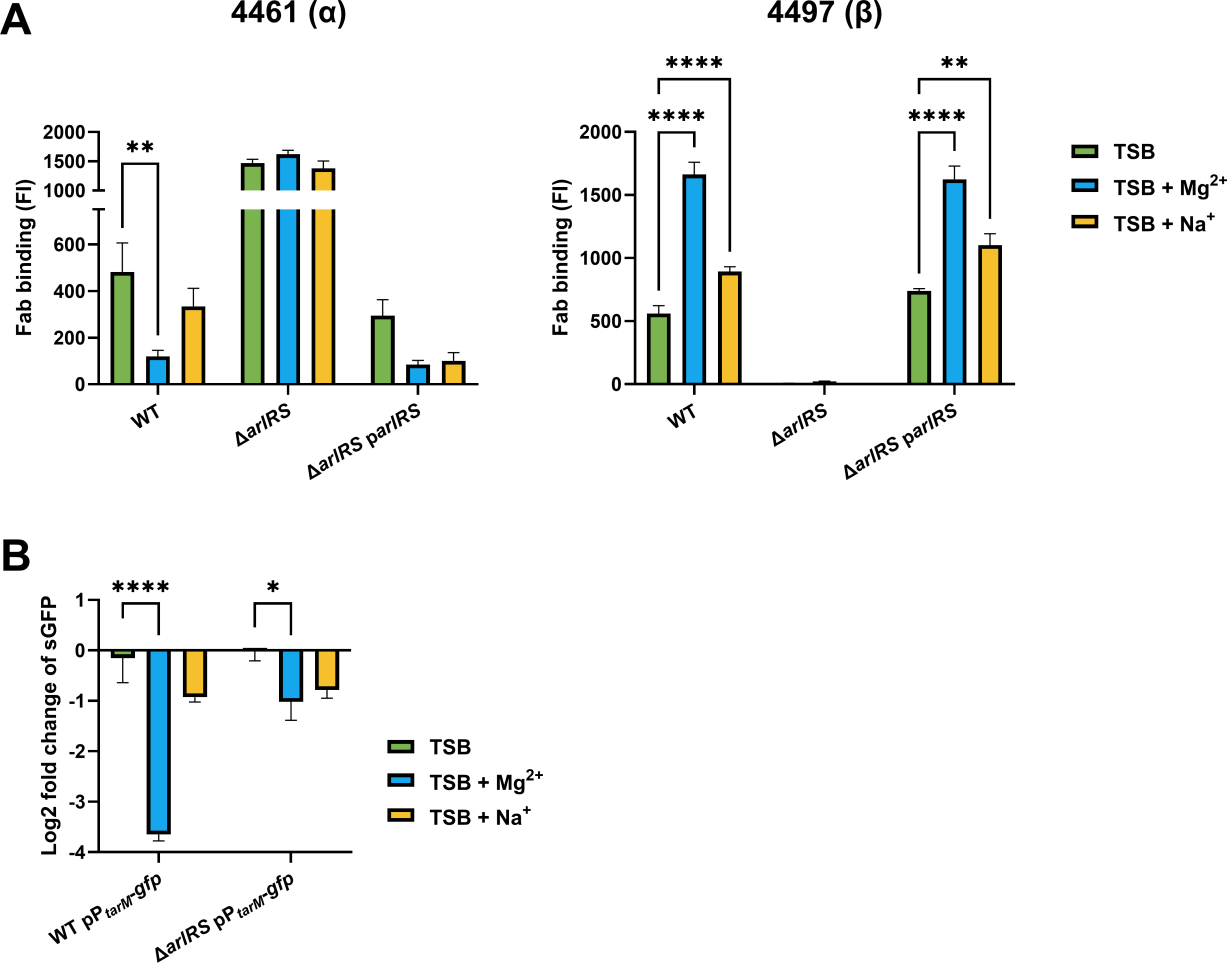
Mg^2+^ drives WTA glycoswitch towards β1,4-GlcNAc and is ArlRS dependent. (**A**) Levels of WTA α1,4- and β1,4-GlcNAc, quantified by binding of Fab fragments 4461 (α) and 4497 (β) to WT, Δ*arlRS* and Δ*arlRS* p*arlRS* when grown in TSB or TSB supplemented with 200 mM Mg^2+^ or 200 mM Na^+^. Data are represented as fluorescence intensity (FI) as analyzed by flow cytometry. (**B**) Promoter activity of *tarM*, displayed as log2 fold change in fluorescence intensity of sGFP in WT pP*_tarM_-gfp* or Δ*arlRS* pP*_tarM_-gfp* bacteria grown in TSB supplemented with 200 mM Mg^2+^ or 200 mM Na^+^ compared to regular TSB. Data represent three biological replicates ±SEM. Statistical significance was determined using two-way ANOVA with Bonferroni statistical hypothesis testing to correct for multiple comparisons. The mean of each column was compared to the mean of TSB marking only significant comparisons. *P < 0.05, **P < 0.01, ****P < 0.0001.

High salt concentrations are known to induce a shift in *S. aureus* WTA glycosylation patterns from predominantly α1,4-GlcNAc to β1,4-GlcNAc decoration (21). The high salt medium previously used contained both Mg^2+^ (15 g/L MgCl_2_ = 158 mM) and Na^+^ (41 g/L NaCl = 702 mM) to induce a stress response. Previously, ArlRS activity was linked to the presence of the divalent cation Mg^2+^ (29). To investigate whether the salt-induced WTA glycoswitch depended on Mg^2+^ or Na^+^, WT and Δ*arlRS* were grown in TSB and TSB supplemented with either 200 mM Mg^2+^ (TSB + Mg^2+^) or 200 mM Na^+^ (TSB + Na^+^). These salt concentrations did not alter bacterial growth (Fig. S1B and C). WTA glycosylation was assessed by binding of Fabs 4461 and 4497. Corresponding to previous findings, Mg^2+^ strongly affected WTA glycosylation levels in WT and complemented Δ*arlRS* p*arlRS* bacteria, with a significant decrease of 4461 (α1,4-GlcNAc) Fab binding and concomitant increase in 4497 (β1,4-GlcNAc) Fab binding compared to growth in TSB (Fig. 4A). By contrast, the addition of Na^+^ only slightly increased levels of β1,4-GlcNAc, but had no significant effect on α1,4-GlcNAc levels (Fig. 4A). No differences in α1,4- and β1,4-GlcNAc levels were observed in Δ*arlRS* when grown in TSB supplemented with Mg^2+^ or Na^+^ compared to growth in TSB only. These results suggest that salt-induced glycoswitching is driven by Mg^2+^, but not Na^+^, and is ArlRS-dependent under the growth conditions used. We next assessed whether the Mg^2+^-induced WTA glycoswitch was caused by changes in *tarM* promoter activity using our sGFP transcriptional reporter system. Mg^2+^ strongly reduced *tarM* promoter activity in WT but not in the Δ*arlRS* mutant (Fig. 4B). These results suggest that Mg^2+^ signals through ArlRS to repress *tarM* promoter activity, which results in increased β1,4-GlcNAc levels.

### TCS ArlRS represses *tarM* expression through MgrA

**Fig 5:**
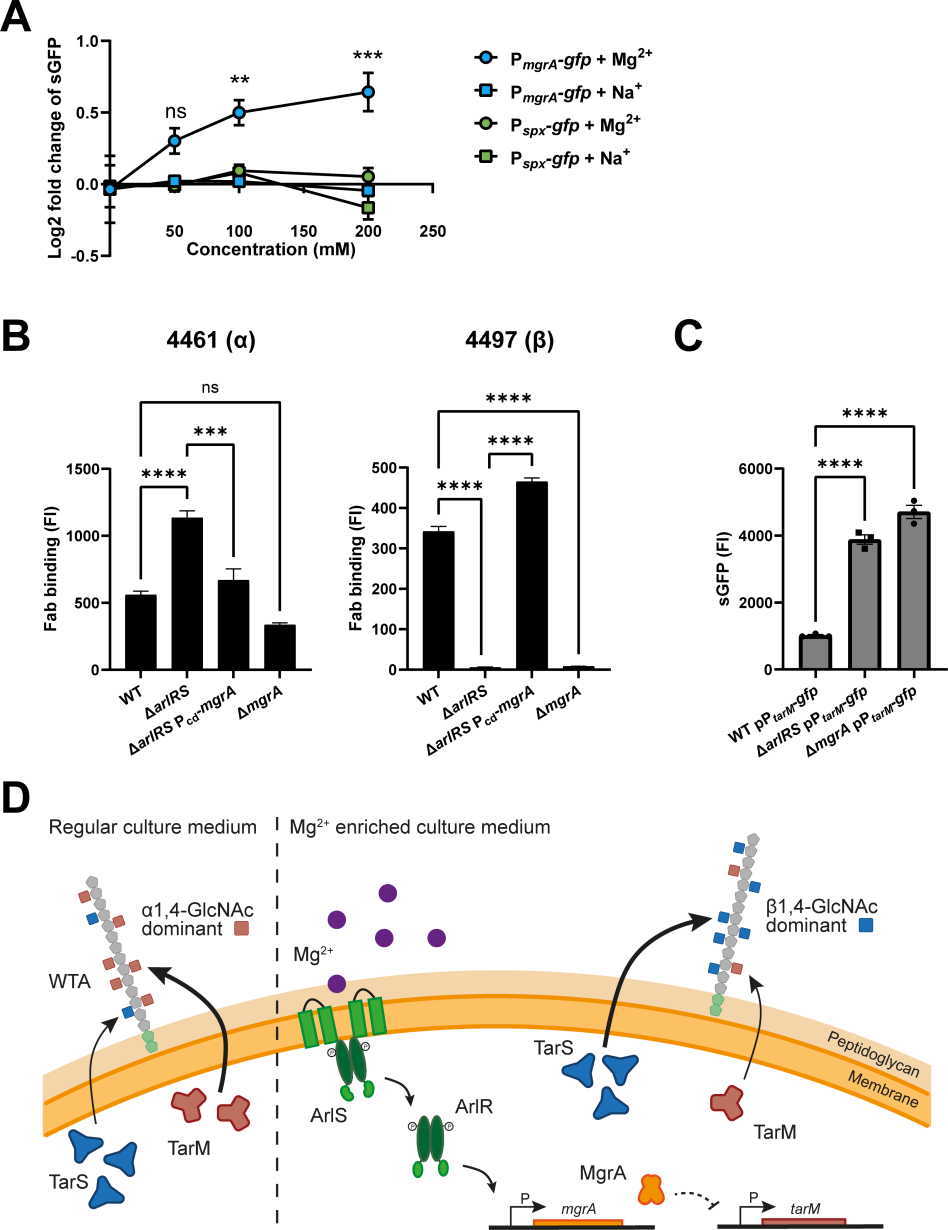
ArlRS represses *tarM* expression through MgrA. (**A**) Promoter activity of *mgrA* and *spx*, displayed as log2 fold change in fluorescence intensity of sGFP in WT pP*_mgrA_-gfp* or WT pP*_spx_-gfp* bacteria grown in TSB supplemented with Mg^2+^ or Na^+^ at various concentrations. (**B**) Levels of α1,4- and β1,4-GlcNAc, quantified by binding of Fab fragments 4461 (α) and 4497 (β) to WT, Δ*arlRS*, Δ*arlRS* P_Cd_-*mgrA* and Δ*mgrA* of *S. aureus* MW2. Data are represented as fluorescence intensity (FI) as analyzed by flow cytometry. (**C**) sGFP fluorescence as a measure for promoter activity of *tarM* in WT pP*_tarM_-gfp*, Δ*arlRS* pP*_tarM_-gfp,* and Δ*mgrA* pP*_tarM_-gfp* bacteria as analyzed by flow cytometry and represented as fluorescence intensity (FI). Data represent three biological replicates ±SEM. Statistical significance was determined using one- (**B, C**) and two-way (**A**) ANOVA with Bonferroni statistical hypothesis testing to correct for multiple comparisons. **P < 0.01, ***P < 0.001, ****P < 0.0001, ns = not significant. (**D**) Schematic overview of the proposed pathway for ArlRS-regulated WTA glycoswitching. Mg^2+^ activates ArlRS, inducing expression of *mgrA*. MgrA in turn either directly or indirectly represses *tarM* expression, allowing TarS to decorate WTA with predominantly β1,4-GlcNAc.

In previous research, *tarM* was not identified as part of the ArlRS regulon (41). However, regulation could be indirect since ArlRS tightly regulates the expression of the global transcriptional regulators *mgrA* and *spx* by binding to a sequence motif in the promoter region. In turn, MgrA and Spx control the expression of numerous downstream genes (33, 41). To investigate the link between ArlRS and *tarM* regulation, we analyzed the activity of the *spx* and *mgrA* promoters with the sGFP-reporter system in both WT and the Δ*arlRS* mutant strain. sGFP fluorescence was measured after overnight culture in TSB containing 50, 100, or 200 mM of Mg^2+^ or Na^+^ and was compared to fluorescence of bacteria grown in TSB only. We observed a dose-dependent increase in *mgrA* promoter activity in response to Mg^2+^, but not Na^+^ (Fig. 5A). By contrast, the *spx* promoter did not respond to the addition of Mg^2+^ or Na^+^ in the medium (Fig. 5A). These observations suggest that Mg^2+^ drives the WTA glycoswitch though ArlRS-dependent activation of *mgrA*.

We postulated that MgrA is part of the regulatory network of ArlRS-dependent WTA glycosylation. To confirm this experimentally, the native *mgrA* promoter was exchanged with a cadmium-inducible promoter in the Δ*arlRS* mutant (Δ*arlRS* P_Cd_-*mgrA*). Importantly, the Δ*arlRS* P_Cd_-*mgrA* strain expressed *mgrA* and *tarM* mRNA at levels comparable to those in WT and Δ*arlRS* p*arlRS* (Fig. S2). Chromosomal exchange of P_Cd_-*mgrA* restored WTA α1,4-GlcNAc and β1,4-GlcNAc glycosylation to WT levels (Fig. 5B). Remarkably, while the Δ*mgrA* mutant showed the absence of β1,4-GlcNAc, it maintained normal levels of α1,4-GlcNAc compared to WT (Fig. 5B). These results indicate that MgrA is involved in the ArlRS-dependent WTA glycosylation.

To investigate whether the deletion of *mgrA* also influences *tarM* promotor activity, we transformed the pP*_tarM_-gfp* plasmid in Δ*mgrA*, creating Δ*mgrA* pP*_tarM_-gfp* and compared its sGFP levels with WT and Δ*arlRS*. Similar to Δ*arlRS*, Δ*mgrA* showed a significantly increased *tarM* promoter activity compared to WT (Fig. 5C). Collectively, these results indicate that ArlRS regulates WTA glycoswitching through the transcription factor MgrA, which represses *tarM* transcription. The proposed pathway is visualized in Fig. 5D.

### ArlRS affects β1,4-GlcNAc-dependent functions as langerin binding and phage infection

To assess the functional consequences of ArlRS-dependent glycoswitching, we explored its effects on β1,4-GlcNAc-mediated processes, including antibiotic susceptibility, langerin binding, and phage susceptibility.

**Fig 6:**
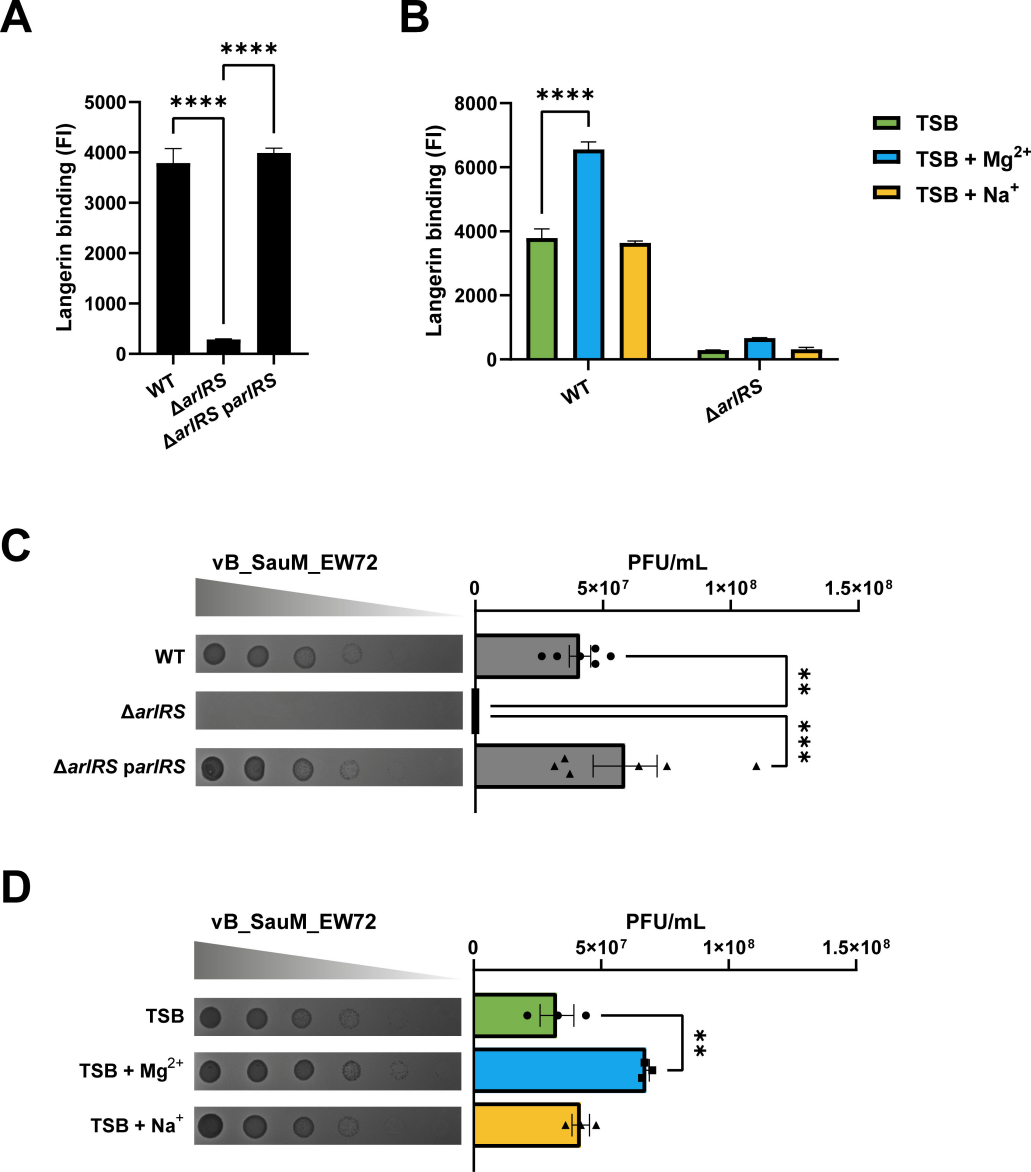
ArlRS activity is required for langerin binding and phage-mediated lysis. (**A, B**) Binding of recombinant langerin receptor to overnight cultures of (**A**) WT, Δ*arlRS,* and Δ*arlRS* p*arlRS* bacteria or (**B**) WT and Δ*arlRS* grown in regular TSB or TSB supplemented with 200 mM Mg^2+^ or Na^+^. Data are represented as fluorescence intensity (FI) as analyzed by flow cytometry from three biological replicates. (**C, D**) Phage dilutions of vB_SauM_EW72 were spotted on a lawn of (**C**) WT, Δ*arlRS* or Δ*arlRS* p*arlRS* bacteria or (**D**) WT bacteria grown in TSB or TSB supplemented with 200 mM Mg^2+^ or Na^+^. Representative images are shown of the formed plaques. PFU/mL was determined from three or six biological replicates. Data are shown as mean ± SEM. Statistical significance was determined using one- (**A, C, D**) and two-way (**B**) ANOVA with Bonferroni statistical hypothesis testing to correct for multiple comparisons. For panels B and D, the mean of each column was compared to the mean of TSB marking only significant comparisons. **P < 0.01, ***P < 0.001, ****P < 0.0001.

We have previously shown that langerin, a C-type lectin receptor of Langerhans cells, recognizes *S. aureus* WTA β1,4-GlcNAc, which subsequently initiates an inflammatory response in the skin (11). We hypothesized that ArlRS could affect langerin binding, thereby impacting the first response of Langerhans cells. Using recombinant FITC-labeled constructs of the extracellular domains of langerin, we observed that langerin binding was greatly diminished in bacteria lacking *arlRS* compared to WT bacteria (Fig. 6A). This phenotype could be restored by complementing *arlRS* (Fig. 6A). Correspondingly, langerin binding to WT bacteria could be further enhanced by supplementing the culture medium with Mg^2+^, but not Na^+^ (Fig. 6B). In line with our hypothesis, langerin binding to the Δ*arlRS* mutant was not affected by the addition of Na^+^ or Mg^2+^.

WTA glycosylation also affects phage infection as some phages, for example, Stab20 and Stab20-like phages such as vB_SauM_EW72 (22), require WTA β-GlcNAc moieties to bind to and subsequently infect *S. aureus* (16, 17). We therefore tested the phage susceptibility of our strains. As shown previously (22), we observed phage infection of WT bacteria, with clearly visible plaques that averaged 4*10^7^ PFU/mL (Fig. 6C; Fig. S3). However, no plaques were observed in the Δ*arlRS* mutant, indicating a 7-log difference in infectivity (Fig. 6C). Supplementation with Mg^2+^, but not Na^+^, enhanced infection by some phages in WT bacteria, although this effect was relatively minor (Fig. 6D; Fig. S4).

Finally, previous research showed that β1,4-GlcNAc decoration contributes to β-lactams resistance in MRSA (3). We confirmed that the Δ*arlRS* mutant was more sensitive to oxacillin, but remained resistant to penicillin, compared to WT and Δ*arlRS* p*arlRS* (Fig. S5). Together, langerin binding, phage infection data, and antibiotic susceptibility assays demonstrate that ArlRS-dependent glycoswitching has biologically relevant consequences in host and phage interaction and antibiotic resistance.

## DISCUSSION

In this study, we have shown that the TCS ArlRS, through the transcriptional regulator MgrA, plays an essential role in regulating the specific GlcNAc decoration of the cell wall glycopolymer WTA, affecting phage infection, langerin binding, and antibiotic resistance. In one-third of *S. aureus* isolates, WTA is not only decorated with β1,4-GlcNAc through the enzyme TarS but is additionally co-decorated with α1,4-GlcNAc through the accessory enzyme TarM (12). These TarM/TarS-positive bacteria can thereby regulate WTA glycosylation to switch between α1,4- or β1,4-GlcNAc-dominant profiles, attuning them to the environmental conditions. The capacity to dynamically regulate the WTA glycoprofile potentially provides a significant survival advantage to *S. aureus* in specific host niches or for antibiotic susceptibility. Moreover, since WTA is a promising target for various alternative treatment options such as vaccines, antibody-dependent therapies, and phage therapy, it is important to understand how WTA glycoswitching is regulated to align antimicrobial strategies with WTA glycosylation profiles during infection.

By screening 1,920 transposon mutants, we identified 230 mutants with a divergent α1,4- or β1,4-GlcNAc WTA glycosylation profile. We identified *tarS* and *tarM* as independent hits in the respective antibody screens confirming the validity of the approach. No other genes in the WTA biosynthesis pathway were identified since these mutants are not included in the NTML. Fifteen of the identified genes had regulatory functions including the three TCS ArlRS, AgrCA, and GraRS. Using a panel of isogenic TCS mutants, we confirmed the role of ArlRS in regulating α1,4- and β1,4-GlcNAc WTA glycosylation. A lack of ArlRS resulted in a complete loss of β1,4-GlcNAc, with concomitantly increased WTA α1,4-GlcNAc decoration. Moreover, the role of ArlRS in regulating the WTA α1,4/β1,4-GlcNAc ratio was non-redundant since the WTA glycosylation phenotype could be restored by plasmid-based *arlRS* complementation in a ΔXV mutant, which lacks all 15 non-essential TCS. We were unable to confirm the contribution of *graRS* and *agrCA* in the regulation of the WTA glycosylation profile, although both TCS have been implicated in the regulation of *tarM* expression (22, 28). This may be explained by the use of different strain backgrounds since the NTML is constructed in the *S. aureus* USA300 JE2 background. The TCS deletion mutants used in this study were in *S. aureus* USA400 MW2 and the previous report on the role of AgrCA in the regulation of WTA glycosylation used *S. aureus* Newman (22). However, strain-specific effects of *agr*-dependent regulation have been previously documented (23). In addition, glycosylation differences were only observed when stimulating the AgrCA pathway with autoinducing peptides, but not when inactivating this pathway through deletion of *agrA* (22). Therefore, the effect of AgrCA on WTA glycosylation profiles may depend on the activity and regulation of the quorum-sensing system of the particular strain and the experimental conditions.

Using a *tarM*-specific GFP-promoter and mRNA expression analysis, we showed that ArlRS-mediated regulation of the WTA glycosylation profile resulted from increased expression of *tarM*, without any apparent changes in *tarS* expression. Similarly, re-analysis of *S. aureus* transcriptome data generated in 44 *in vitro* and *in vivo* mimicking conditions, demonstrated that *tarM* expression is more dynamically regulated, while *tarS* levels remained constant at a relatively high level in all experimental conditions (24, 37). Since the genes of the *tar* WTA biosynthesis operon, including *tarS*, feature similarly stable expression (24, 37), regulation of the WTA glycoprofile may not be possible through regulation of *tarS* without affecting general WTA turnover. Instead, it could take place through regulation of the accessory *tarM* gene located outside of the *tar* operon. As a result of increased transcription, the increased levels of TarM likely outcompete TarS at a functional level due to its higher enzymatic activity (42, 43). *In vitro* biochemical assays with different ratios of recombinant TarM and TarS may be able to confirm this enzymatic competition in the future.

We were able to reduce *tarM* expression and restore WTA β1,4-GlcNAc glycosylation in the Δ*arlRS* mutant by overexpressing *mgrA*, demonstrating that ArlRS-mediated regulation of *tarM* expression requires *mgrA* and not *spx*. MgrA itself was not identified in our screen since this gene is not included in the NTML. Unexpectedly, the deletion of *mgrA* did not completely phenocopy the *arlRS* mutant. Although the *mgrA* mutant showed a complete loss of β1,4-GlcNAc similar to the *arlRS* mutant, *mgrA* deletion did not result in a significant concomitant increase in WTA α1,4-GlcNAc decoration. It is already known that the phenotypes of *arlRS* and the *mgrA* deletion strains show overlap due to overlapping regulons (31, 34, 41, 44), but *tarM* is not part of either regulon (41, 45). A similar observation is made in a recent paper, in which MgrA is identified as the regulator of the *ssc* genes encoding for a novel *S. aureus* secondary glycopolymer, but no canonical *mgrA*-binding site could be found (46). Alternatively, the deletion of *mgrA* may interfere with other regulatory pathways. Indeed, MgrA represses the expression of the transcription factors SarV and AtlR (33), which may indicate that MgrA regulates *tarM* expression indirectly. Therefore, the molecular mechanisms by which MgrA represses *tarM* remain elusive. Furthermore, despite recent advances in RNA sequencing or prediction of transcription factor binding sequences, our observations stress the importance of combining phenotypic screening with experimental work to confirm direct gene regulation (33, 47).

Shifts in WTA glycosylation profiles can be induced by general stress, such as high salt concentrations (21). The medium previously used contained both high Na^+^ and Mg^2+^ concentrations. We first demonstrated that salt-induced effects on WTA glycoprofiles were induced by high levels of Mg^2+^ but not Na^+^. We next showed that these Mg^2+^-induced changes in the WTA glycoprofile depended on the repression of *tarM* promoter activity, which required the ArlRS TCS. Our data extend earlier research on the link between ArlRS, Mg^2+^, and WTA, where it was shown that ArlRS and Mg^2+^ repressed the *dlt* operon (29), which decorates WTA with D-alanine residues. In addition to transcriptional changes, biochemical studies have shown that Mg^2+^ acts as a co-factor to increase TarS enzymatic activity, which in our conditions may have additionally boosted β1,4-GlcNAc modifications (43). Unexpectedly, we also detected a slight increase in WTA β1,4-GlcNAc levels when TSB was supplemented with Na^+^. However, Na^+^ did not affect *tarM* or *mgrA* promoter activity. Therefore, these Na^+^-induced effects most likely involved a different pathway independent of ArlRS or MgrA.

Mg^2+^ is an essential mineral for many basic biological processes and one of the four most common cations in the human body (36). Ninety-nine percent of the total amount of Mg^2+^ is found in bones, muscle, and soft tissue (48). The remaining Mg^2+^ is present in red blood cells and serum (48, 49). *S. aureus* can naturally infect the skeletal organ (50). This Mg^2+^-enriched niche seems to favor chronic infections and induce biofilm formation of *S. aureus* (51, 52). Although in our study Mg^2+^ clearly had a great influence on *S. aureus*, there are no studies yet that analyzed the complete gene set regulated by Mg^2+^, as has been done for *Streptococcus pyogenes* (53). In *S. pyogenes*, CovS of the streptococcal TCS CovRS has been identified as the sensor for Mg^2+^ and has 32% sequence identity with ArlS. As no dedicated activation signal for ArlRS has been identified, we propose that Mg^2+^ may be a primary stressor for the ArlRS TCS in *S. aureus*. These findings suggest an important role of metal ion availability in the *S. aureus* WTA glycoswitching. Moreover, it highlights how *S. aureus* could undergo phenotypic WTA modifications in environments with high Mg^2+^ concentrations such as bone, muscle, and soft tissue through the sensing and transcriptional regulation of ArlRS and MgrA.

ArlRS plays a role in several host-pathogen interactions (30–35), including several animal models (31, 33, 34, 44). To probe the potential functional consequences of ArlRS-mediated WTA glycoswitching for biologically relevant interactions, we performed studies to investigate the interaction with human langerin and the impact on infection by β1,4-GlcNAc-dependent phages. Langerin is a pattern recognition receptor unique to innate Langerhans cells that specifically binds staphylococcal WTA β-GlcNAc and is hindered by the co-presence of α1,4-GlcNAc. This interaction acts as the first response in the defense against *S. aureus* in the skin through the induction of skin inflammation specifically neutrophil recruitment (11). As expected, the increased β1,4-GlcNAc levels in response to high Mg^2+^ and dependence on ArlRS activation resulted in increased Langerin binding. How the conditions in human skin including the concentration of Mg^2+^ would affect ArlRS activation and subsequent WTA glycosylation profiles is currently unknown but warrants further investigation.

With regard to phage infection, the effect of WTA glycoswitching is a double-edged sword. Whereas bacteria may profit from phages to gain favorable genes *via* horizontal gene transfer (4), lytic phages can also kill bacteria (54). WTA decoration with GlcNAc is required for successful infection by siphophages, podophages, and some myophages (5, 15–17), but some phages require GlcNAc to be present in a specific configuration (55). In this study, all used phages depend on the WTA β1,4-GlcNAc modification to infect and lyse *S. aureus* (22). Consequently, it was expected that phage infection was completely abolished in the Δ*arlRS* mutant lacking β1,4-GlcNAc. This is consistent with a previous observation that after co-culture, *S. aureus* bacteria resistant to the β1,4-GlcNAc-dependent podophage carried an inactivating mutation in ArlRS (56). In contrast to our finding with langerin and Fab binding experiments, the influence of Mg^2+^ on phage infection was modest. This may be due to the long infection period on the agar plates that did not contain extra Mg^2+^. Nevertheless, our results still suggest that Mg^2+^ concentrations influence *S. aureus* phage infection. This has relevance for choosing the right phage therapy, as an isolate may be different with regard to WTA glycosylation depending on the location of the infection, such as a osteomyelitis versus endocarditis. Therefore, accurate insight in WTA glycosylation could play a crucial role in preventing future phage therapy failures.

In summary, we identified the critical importance of ArlRS in the dynamic regulation of the WTA GlcNAc decoration in *S. aureus*. ArlRS was activated by high concentrations of Mg^2+^ and subsequently activated MgrA and upregulated *tarM* expression, resulting in WTA glycoswitching from a β1,4-GlcNAc-dominated to an α1,4-GlcNAc-dominated profile. As WTA glycosylation patterns impact many biologically relevant and infection-relevant interactions, more research is needed to determine differential GlcNAc levels of *S. aureus* isolates in specific tissues such as bone and skin, common sites of (chronic) *S. aureus* infections. This is especially important since *in vitro* testing of GlcNAc levels in clinical isolates after routine culture or screening for gene presence does not correlate with *in vivo* WTA glycosylation patterns. These explorations could help prioritize the design of new antimicrobial strategies such as vaccines and phage therapy targeting specific WTA glycan modifications.

## MATERIALS AND METHODS

### Bacterial strains and culture conditions

All *S. aureus* cultures were grown in Tryptic Soy Broth (TSB, Oxoid) overnight at 37°C with continuous shaking. The Nebraska Transposon Mutant Library (NTML; 1,920 arrayed mutants of *S. aureus* strain JE2, harboring both *tarM* and *tarS*) (38) was grown in the presence of 5 µg/mL erythromycin (Sigma). *S. aureus* complemented strains were grown with the addition of 10 µg/mL erythromycin or 10 µg/mL chloramphenicol (Sigma), depending on the resistance marker used. When required, TSB was enriched with MgCl_2_ (TSB + Mg^2+^, Merck) or NaCl (TSB + Na^+^, Merck) in a concentration of 200 mM unless stated otherwise. *Escherichia coli* strain DC10B (57) was grown at 37°C with continuous shaking in Lysogeny broth (LB) supplemented with 100 µg/mL ampicillin (Sigma) when appropriate. All strains used in this study are listed in Table S2.

### Analysis of *tarM* and *tarS* mRNA transcription levels

The transcriptome of *S. aureus* HG001 grown in 44 different experimental conditions was analyzed in earlier research (24). The transcript levels of *tarS* (SAOUHSC_00228) and *tarM* (SAOUHSC_00973) were downloaded from the AureoWiki repository (37). Averages of biological replicates were calculated and plotted, comparing *tarM* and *tarS* expressions in all different growth conditions. Statistical difference in the variance of the transcript levels was calculated using an F test with GraphPad Prism 10.2.0.

### NTML screen for WTA α1,4- and β1,4-*N*-acetylglucosamine glycosylation levels

The protocol for immunoblotting was based on Weber et al. (58) with some modifications. Each of the 1,920 NTML (38) transposon mutants was inoculated from the frozen stock into a single well of a 96-well round bottom plate (Sigma) containing TSB with 5 µg/mL erythromycin. The next day, 2 µL of the overnight culture was spotted in duplicate onto Tryptic Soy Agar (TSA, Oxoid) plates containing 5 µg/mL erythromycin. Cultures of JE2 WT and the markerless deletion mutants of *ΔtarMS*, Δ*tarM*, and Δ*tarS* in JE2 background were spotted on TSA plates as a control. All plates were incubated overnight and bacteria were transferred from each plate to a 0.45 µm nitrocellulose membrane (Biorad). Each membrane was dried for 20 min and subsequently washed with demineralized water and Tris-buffered saline (TBS). The membranes were blocked for 1 hour with TBS supplemented with 1% Tween20 (TBST, Fisher Scientific) and 5% bovine serum albumin (BSA; Sigma) and washed with TBST. The glycosylation of each transposon mutant was analyzed using the specific Fab clones 4461 (α1,4-GlcNAc) and 4497 (β1,4-GlcNAc) (59) by incubating the membranes for 1 hour with either 0.5 µg/mL 4461 or 4497 Fabs in TBST. After washing with TBST, the membranes were incubated for 1 hour with a 1:1,000 dilution of Goat Fab Anti-Human IgG Kappa-AP (Southern Biotech) and washed again with TBST. Fab staining, representing WTA glycosylation, was visualized using Vector blue AP Substrate (Vector laboratory). The reaction was stopped with a quick wash with demineralized water and blots were imaged using a Uvitec Platinum V10 imager. Differential glycosylation patterns were assessed by visual comparison of the overall staining intensity of mutants on a single blot.

### Bacterial cloning

The deletion mutants Δ*tarM*, Δ*tarS* and the double mutant Δ*tarMS* in JE2 background were previously generated (60). MW2 wild-type (WT) bacteria and a panel of cognate TCS deletion mutants were previously generated (25, 44). Complementation mutants of ΔXV and Δ*arlRS* were constructed using the pCN51 plasmid under the control of a cadmium promoter, yielding ΔXV p*arlRS* and Δ*arlRS* p*arlRS* (25). Cadmium was not used for the expression of genes as the leaky expression on this plasmid was sufficient to restore the phenotype (25). The *arlRS* deletion mutant complemented with *mgrA* (Δ*arlRS* P_Cd_-*mgrA*) was constructed by Burgui et al. (44) *via* allelic exchange of the *mgrA* promoter with the cadmium inducible promoter. As this complementation is chromosomal rather than plasmid-based, 5 µM cadmium was added to the culture medium to reach sufficient ArlRS-independent expression of *mgrA*.

### Analysis of α1,4- and β1,4-*N*-acetylglucosamine glycosylation levels

WTA glycosylation with either α1,4- or β1,4-GlcNAc was analyzed using specific Fab fragments. Overnight cultures in TSB with or without appropriate antibiotics were resuspended in PBS + 0.1% BSA at an optical density 600 nm (OD_600_) of 0.4 and incubated with 3.3 µg/mL Fab fragments 4461 (α1,4-GlcNAc) or 4497 (β1,4-GlcNAc) (59). After washing, bacteria were incubated with 1:200 goat Fab human Kappa IgG Alexa Fluor 647 (Southern Biotech). Bacteria were washed and fixed in 1% paraformaldehyde (PFA, Sigma-Aldrich) and analyzed by flow cytometry (BD FACSCanto II Flow Cytometer, BD Bioscience) using FlowJo version 10 (BD Bioscience).

### Promoter activity analysis using *promoter::gfp* translational fusions

Promoter activity of *tarM*, *mgrA,* and *spx* was analyzed using a fluorescent reporter system with the plasmid pCM29, which contains a *SarA*-P1 promoter upstream of sGFP (40). Specific promoter regions of *mgrA*, *spx* (33, 41), and *tarM* (61) were cloned into pCM29 *via* restriction enzyme digest to replace the *SarA*-P1 promoter. Resulting plasmids were first transformed in *E. coli* DC10B and subsequently transformed into MW2 WT (resulting in WT pP*_mgrA_-gfp*, WT pP*_spx_-gfp*, and WT pP*_tarM_-gfp*), in Δ*arlRS* (resulting in Δ*arlRS* pP*_mgrA_-gfp*, Δ*arlRS* pP*_spx_-gfp*, and Δ*arlRS* pP*_tarM_-gfp*) and in Δ*mgrA* (Δ*mgrA* pP*_tarM_-gfp*). Used primers are listed in Table S3.

sGFP-reporter strains were grown overnight in TSB supplemented with 10 µg/mL chloramphenicol and Mg^2+^ or Na^+^ if appropriate. Cultures were adjusted to an OD_600_ of 0.4 in PBS + 0.1% BSA and fixed with 1% PFA (Sigma-Aldrich). sGFP intensity was analyzed by flow cytometry (BD FACSCanto II Flow Cytometer, BD Bioscience) using FlowJo version 10 (BD Bioscience).

### Absolute quantification of mRNA transcripts by reverse transcriptase (RT)-qPCR

As ArlRS has an extensive influence on the expression levels of many genes, we quantified the absolute number of mRNA copies for *tarM* and *mgrA* to circumvent the use of reference genes. Briefly, pellets of overnight bacterial cultures were resuspended in TRIzol reagent (Invitrogen) and bacterial cells were disrupted using 0.1 mm zirconium sand and 2 mm glass beads using a Magna-lyser for 2 min at 6,000 rpm. Total RNA was extracted using a Direct-zol RNA miniprep-kit (Zymo Research) according to the manufacturer’s protocol followed by a DNase treatment using the Turbo DNA-free kit (Invitrogen). cDNA was synthesized using the Maxima H Minus cDNA Synthesis Master Mix (ThermoFisher) and the absence of genomic DNA was confirmed through no-RT PCRs. RT-qPCR was performed using a standard curve as described by Goerke et al. (62). In short, the genes were amplified with regular PCR using the genomic DNA of *S. aureus* MW2 as a template and primers listed in Table S3. The amplicons were visualized on the gel for specificity, cleaned using GeneJET PCR Purification Kit (Thermo Scientific), and DNA concentration was measured with the Qubit 1X dsDNA HS Assay Kit (Invitrogen) as analyzed with a Qubit 4 Fluorometer. The sequence-specific standard curves of *tarM*, *tarS,* and *mgrA* were then generated using a 10-fold serial dilution (10^2^ to 10^8^ copies/reaction). Quantitative RT-PCR was performed in triplicate on the standard curve and cDNA samples using the PowerTrack SYBR Green Master Mix (Thermo Scientific) in the CFX384 RT-PCR instrument (Bio-Rad) with CFX Maestro 5.0 software. Copy numbers per reaction of samples were determined *via* inter- or extrapolation of the Ct values of cDNA samples.

### Langerin binding

*S. aureus* langerin binding was analyzed as described previously (11, 59). Recombinant FITC-labeled construct of human langerin was kindly provided by Prof. C. Rademacher, University of Vienna, Vienna, Austria (63). In short, overnight cultures were resuspended in TSM buffer (2.4 g/L Tris [Sigma­ Aldrich], 8.77 g/L, NaCl [Merck], 294 mg/L CaCl_2_(H_2_O)_2_ [Merck], 294 mg/L MgCl_2_(H_2_O)_6_ [Merck], containing 0.1% BSA, pH 7) at an OD_600_ of 0.4 and incubated with 20 µg/mL recombinant langerin. Langerin binding was analyzed by flow cytometry (BD FACSCanto II Flow Cytometer, BD Bioscience).

### Phage spot assay

All phages used in this study are listed in Table S2. Stab20 is a lytic myophage within the genus Kayvirus in the subfamily Twortvirinae (64). The Stab20-like myophages have similar receptor-binding proteins as Stab20 (65). Phage susceptibility of *S. aureus* was tested *via* phage spot assay (22) using the double agar overlay method (66). Briefly, overnight cultures were mixed with phage top agar (20 g/L Nutrient Broth No. 2; 3.5 g/L Agar No. 1 supplemented with 10 mM CaCl_2_) and poured over phage base agar (20 g/L of Nutrient Broth No. 2; 7 g/L Agar No. 1 supplemented with 10 mM CaCl_2_) to generate indicator plates. Phage lysates were prepared as described earlier (67) and were diluted to 1 × 10^8^ plaque-forming units (PFU)/mL followed by a 10-fold serial dilution in phage buffer (1 mM MgSO_4_, 4 mM CaCl_2_, 50 mM Tris-HCl, 100 mM NaCl, pH 8.0). Respective dilutions were spotted on top of the indicator plates. After overnight incubation at 37°C, plaques were counted, PFU/mL determined and plates were imaged (BioRad ChemiDoc XRS + imager).

### Statistical analysis

Statistical analysis was performed using GraphPad Prism 10.2.0. The F test was used to compare the variance of two groups, the Student’s *t* test and one- and two-way ANOVA with Bonferroni statistical hypothesis testing to correct for multiple comparisons were used to determine significant differences (*P* < 0.05) between two or more groups. All values are reported as mean with standard error of the mean (SEM) of three biological replicates unless indicated otherwise.

## SUPPLEMENTAL MATERIAL

Fig. S1 mbio.02668-24-s0001.tif Growth curves.

Fig. S2 mbio.02668-24-s0002.tif qPCR.

Fig. S3 mbio.02668-24-s0003.tif Phages.

Fig. S4 mbio.02668-24-s0004.tif Phages and salts.

Fig. S5 mbio.02668-24-s0005.tif Antibiotic susceptibility.

Supplemental Material mbio.02668-24-s0006.docx Supplemental methods, legends for Figures S1-S5 and Table S1, and Tables S2 and S3.

Table S1 mbio.02668-24-s0007.xlsx NTML mutants showing affected 4461 or 4497 binding levels.

ASM does not own the copyrights to Supplemental Material that may be linked to, or accessed through, an article. The authors have granted ASM a non-exclusive, world-wide license to publish the Supplemental Material files. Please contact the corresponding author directly for reuse.
