# Supplementary figures and images for: The two-component system ArlRS is essential for wall teichoic acid glycoswitching in Staphylococcus aureus
Source: mBio. 2024 Nov 29;16(1):e02668-24. doi: 10.1128/mbio.02668-24 (PMC11708061; doi:10.1128/mbio.02668-24)

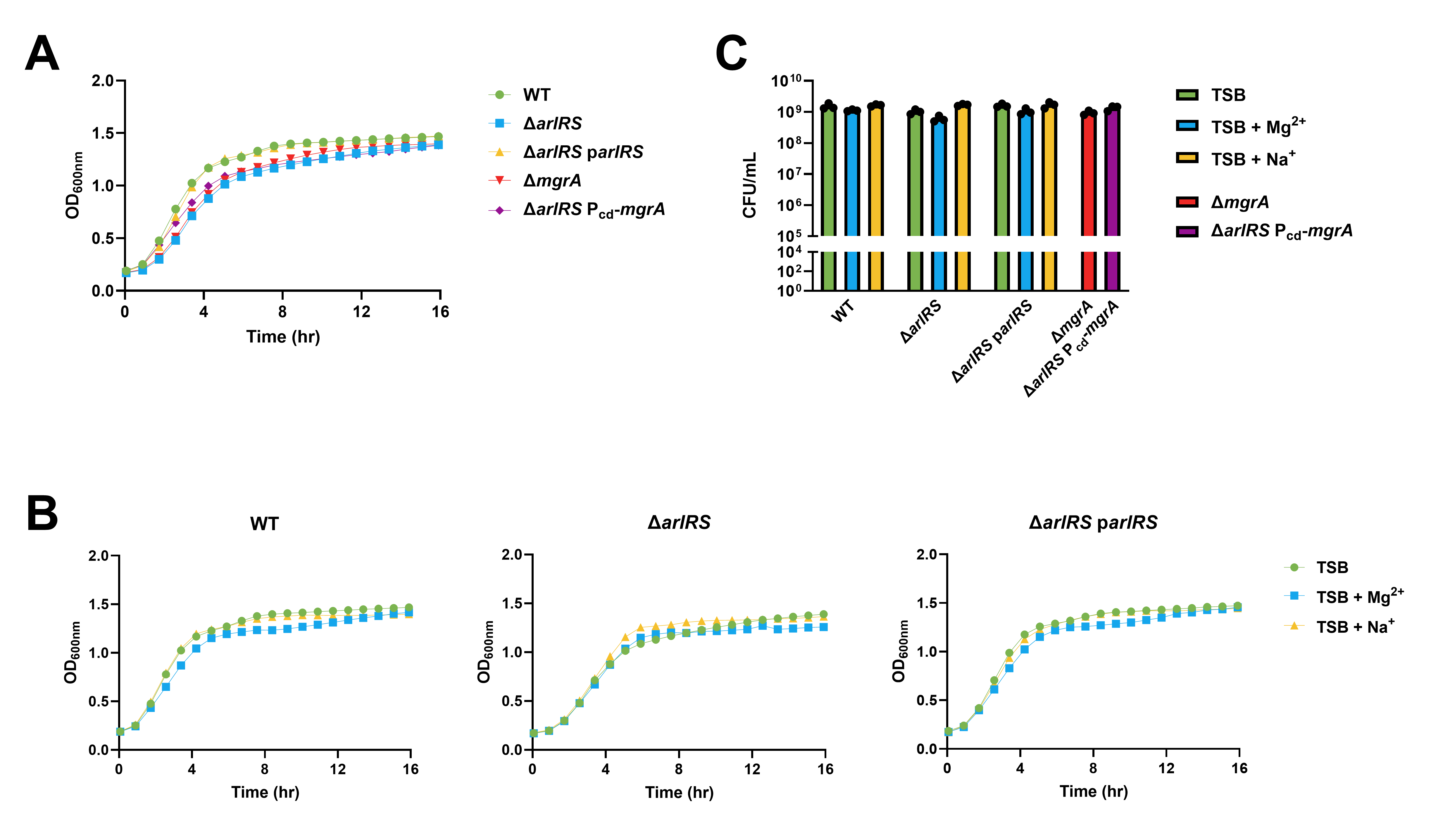

Supplement: Fig. S1 — Growth curves. [file mbio.02668-24-s0001.tif]

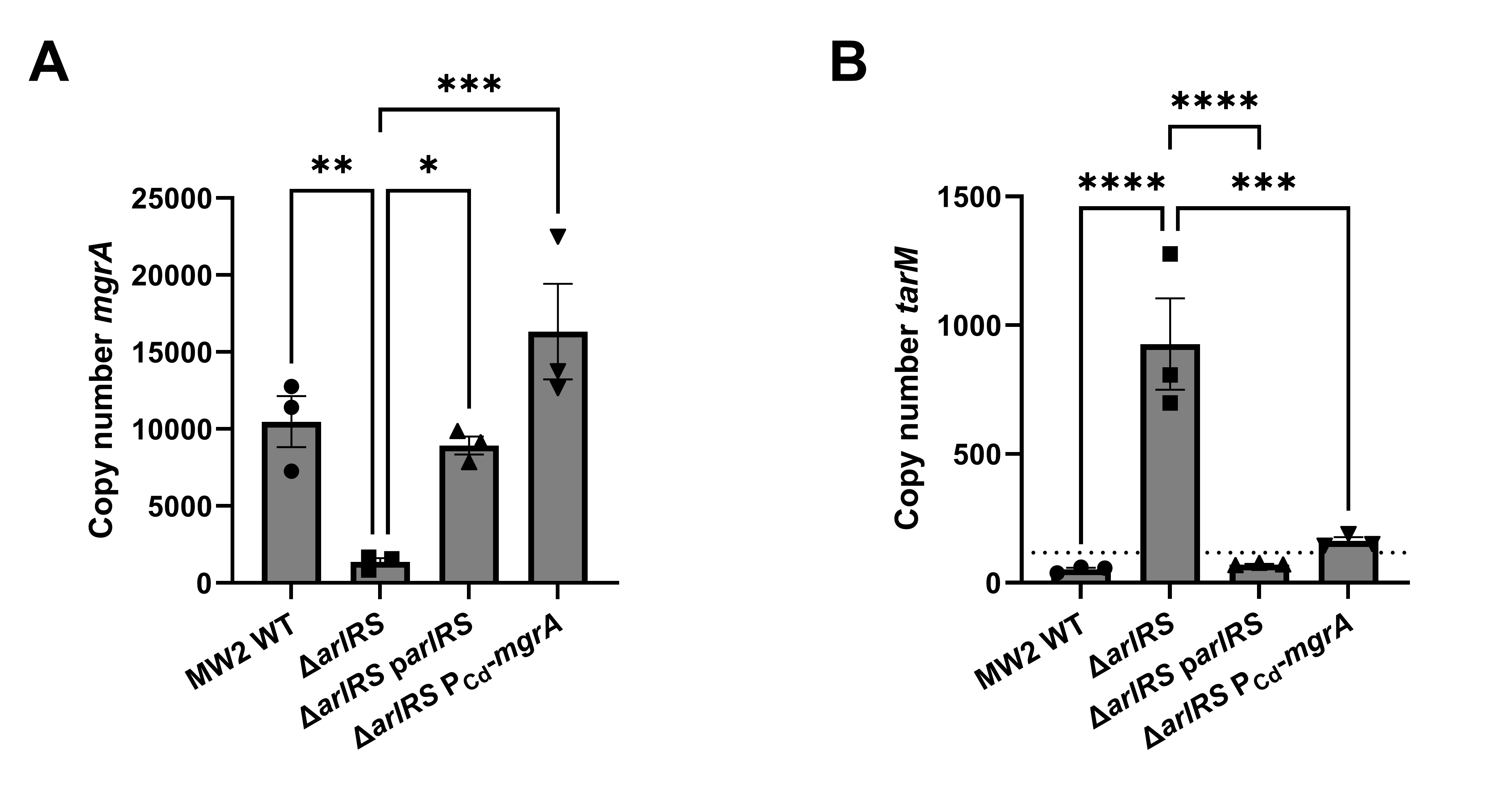

Supplement: Fig. S2 — qPCR. [file mbio.02668-24-s0002.tif]

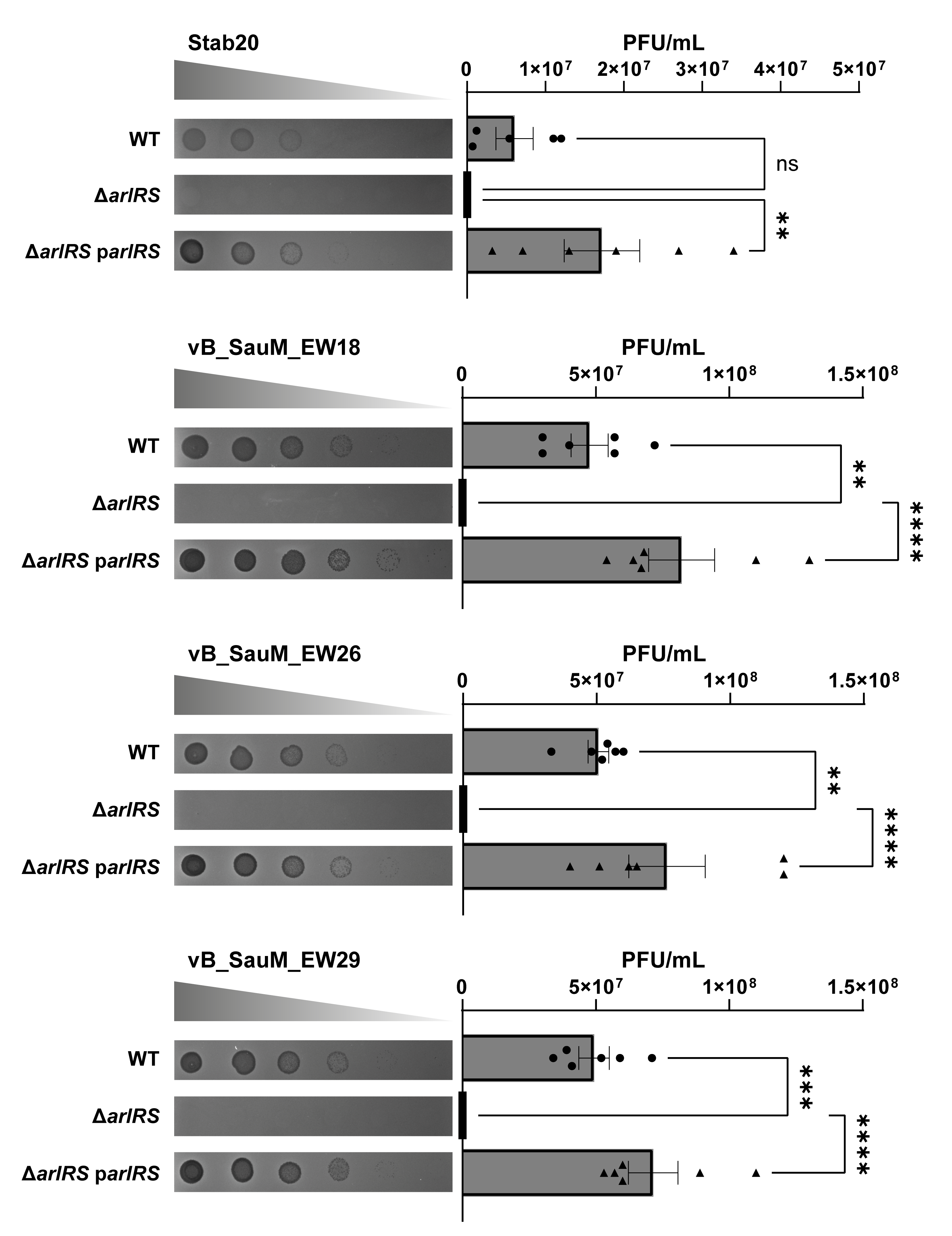

Supplement: Fig. S3 — Phages. [file mbio.02668-24-s0003.tif]

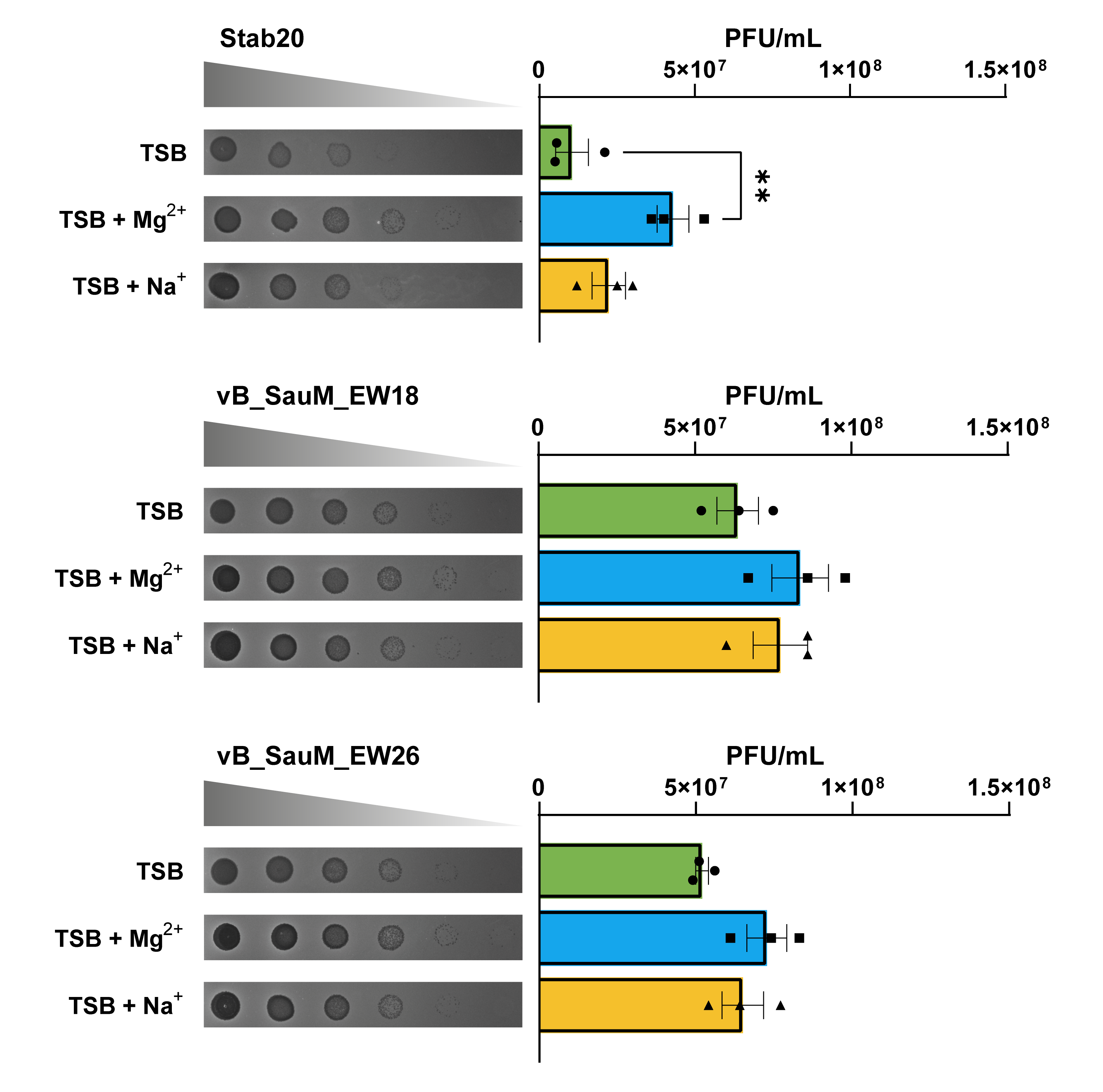

Supplement: Fig. S4 — Phages and salts. [file mbio.02668-24-s0004.tif]

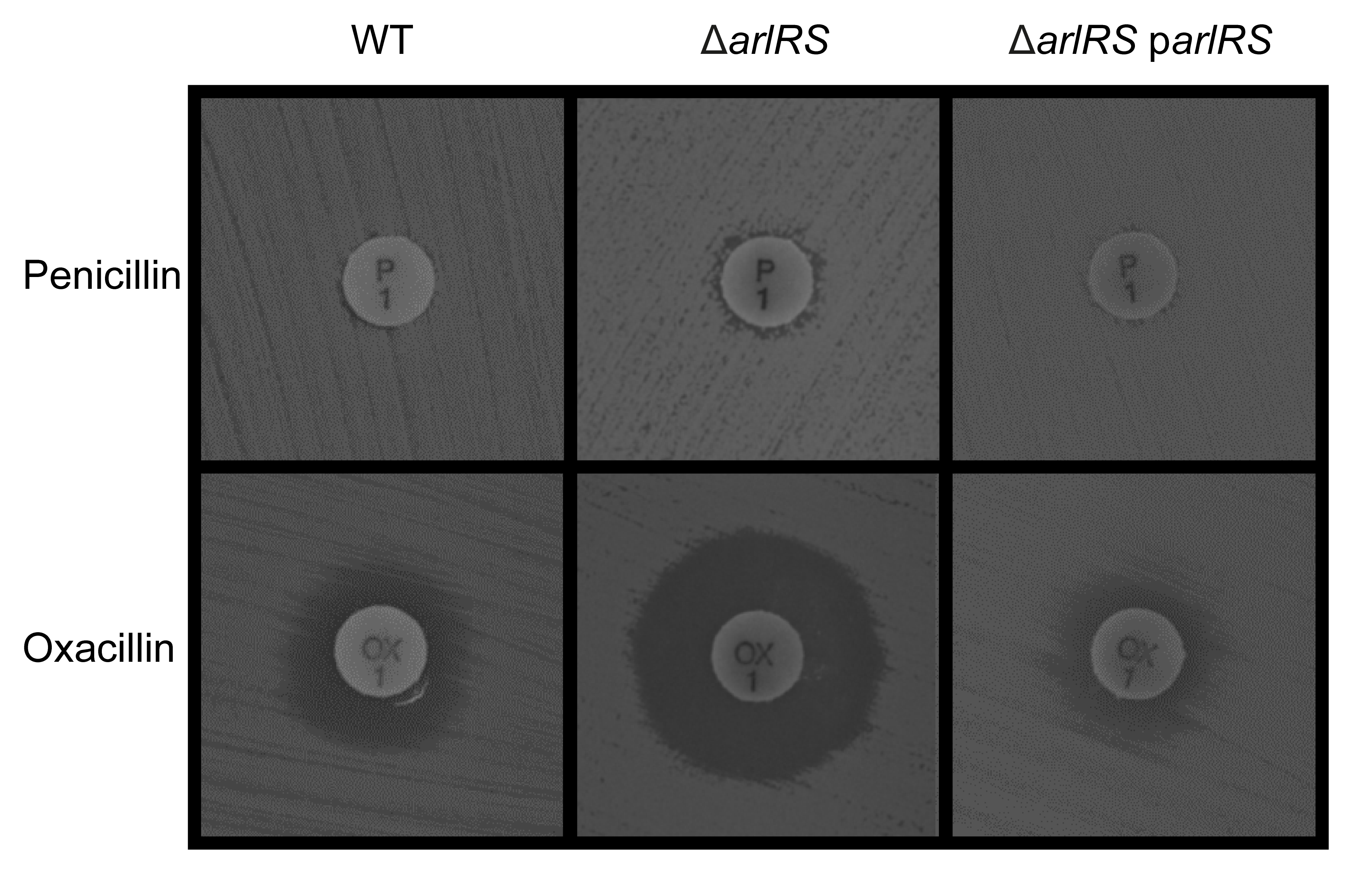

Supplement: Fig. S5 — Antibiotic susceptibility. [file mbio.02668-24-s0005.tif]
