# Supplementary material for: The two-component system ArlRS is essential for wall teichoic acid glycoswitching in Staphylococcus aureus
Source: mBio. 2024 Nov 29;16(1):e02668-24. doi: 10.1128/mbio.02668-24 (PMC11708061; doi:10.1128/mbio.02668-24)
Supplement: Supplemental Material — Supplemental methods, legends for Figures S1-S5 and Table S1, and Tables S2 and S3. [file mbio.02668-24-s0006.docx]

**Supplemental Material**

**Supplemental Methods**

Growth curves

WT, Δ*arlRS*, Δ*arlRS* p*arlRS*, Δ*mgrA* and Δ*arlRS* P_cd_-*mgrA* were grown overnight and sub-cultured the following day to an optical density of 600 nm (OD_600nm_) of 0.4 in TSB supplemented with antibiotics or 200 mM Mg^2+^ or 200 mM Na^+^ where indicated. The early exponential phase bacteria were diluted to OD_600nm_ of 0.025 and OD_600nm_ was measured every 5 minutes over 16 hours with shaking in a Biotek Synergy H1. CFU/mL of overnight cultures were calculated by plating out serial dilutions.

Antibiotic resistance

WT, Δ*arlRS* and Δ*arlRS* p*arlRS* were grown overnight in TSB and diluted in PBS to 0.5 McFarland. Bacteria were inoculated onto TSA plates with a sterile swab and antibiotic discs were placed on top of the agar. Plates were incubated overnight at 37 °C and were imaged using a Uvitec Platinum V10 imager.

**Supplemental Figures**

**Figure S1. Growth characteristics of the bacterial strains.** (A) Growth curves of WT, Δ*arlRS*, Δ*arlRS* p*arlRS*, Δ*mgrA* and Δ*arlRS* P_cd_-*mgrA* in TSB. (B) Growth curves of WT, Δ*arlRS* and Δ*arlRS* p*arlRS* in TSB or TSB supplemented with 200 mM Mg^2+^ or 200 mM Na^+^. (C) Colony forming units (CFU) per mL of overnight cultures of WT, Δ*arlRS* and Δ*arlRS* p*arlRS* in TSB or TSB supplemented with 200 mM Mg^2+^ or 200 mM Na^+^ and of Δ*mgrA* and Δ*arlRS* P_cd_-*mgrA* grown in TSB.

**Figure S2. Expression of *mgrA* and *tarM* in Δ*arlRS* and complemented strains.** (A, B) mRNA copy number of (A) *mgrA* and (B) *tarM* as measured with qPCR in WT, Δ*arlRS* and complemented bacteria Δ*arlRS* p*arlRS* and Δ*arlRS* P_Cd_-*mgrA*. Symbols below the dotted line represent extrapolated values. Data represent three biological replicates ± SEM. First three columns of panel B (WT, Δ*arlRS* and Δ*arlRS* p*arlRS*) are the same data as Figure 3C shown in the main article. Statistical significance was determined using one-way ANOVA with Bonferroni statistical hypothesis testing to correct for multiple comparisons.**p* < 0.05, ***p* < 0.01, ****p* < 0.001, **** *p* < 0.0001.

**Figure S3. ArlRS is required for successful infection of Stab20 and Stab20-like phages in *S. aureus*.** Phage dilutions of Stab20 or the Stab20-like phages vB_SauM_EW18, vB_SauM_EW26 and vB_SauM_EW29 were spotted on a lawn of WT, Δ*arlRS* or Δ*arlRS* p*arlRS* bacteria. Representative images are shown of the formed plaques. PFU/mL were counted from six biological replicates. Data are shown as mean ± SEM. Statistical significance was determined using one-way ANOVA with Bonferroni statistical hypothesis testing to correct for multiple comparisons. ***p* < 0.01, ****p* < 0.001, *****p* < 0.0001, ns=not significant.

**Figure S4. Mg^2+^ enhances infection by Stab20 and Stab20-like phages in *S. aureus*.** Phage dilutions of Stab20 or the Stab20-like phages vB_SauM_EW18 and vB_SauM_EW26 were spotted on a lawn of WT bacteria grown in TSB or TSB supplemented with 200 mM Mg^2+^ or Na^+^. Representative images are shown of the formed plaques. PFU/mL were counted from three biological replicates. Data are shown as mean ± SEM. The mean of each column was compared to the mean of TSB marking only significant comparisons. ***p* < 0.01, ns=not significant.

**Figure S5. β-lactam antibiotic resistance.** Resistance to penicillin and oxacillin of WT, Δ*arlRS* and Δ*arlRS* p*arlRS*.

**Table S1: NTML mutants showing affected 4461 or 4497 binding levels.**

**Table S2. Overview of strains, plasmids and phages used in this study.**

| Strains / plasmids / phages | Description | Reference |
| --- | --- | --- |
| *E. coli* | | |
| DC10B | Host strain for cloning vectors for *S. aureus* | [1] |
| *S. aureus* | | |
| NTML | Nebraska Transposon Mutant Library, containing 1,920 transposon mutants (*S. aureus* JE2) | [2] |
| JE2 WT | Wild type, parent strain of the Nebraska Transposon Mutant Library | [2, 3] |
| Δ*tarMS* | JE2 background with deletion of both *tarM* and *tarS* | [4] |
| Δ*tarM* | JE2 background with deletion of *tarM* | [4] |
| Δ*tarS* | JE2 background with deletion of *tarS* | [4] |
| MW2 WT | Wild type, community-acquired methicillin-resistant *S. aureus* | [5] |
| ΔXV | MW2 background, deletion of all 15 non-essential TCS: ∆*hptRS* ∆*lytSR* ∆*graRS* ∆*saeRS* ∆MW1208-9 ∆*arlRS* ∆*srrAB* ∆*phoPR* ∆*airSR* ∆*vraSR* ∆*agrCA* ∆*kdpDE* ∆*hssRS* ∆*nreBC* ∆*braRS* | [6] |
| ΔXV p*arlRS* | MW2 ∆XV carrying pCN51::*arlRS* plasmid | [6] |
| Δ*agrCA* | MW2 background with deletion of *agrBDCA* (MW1960-3) | [6] |
| Δ*graRS* | MW2 background with deletion of *graRS* (MW0621-2) | [6] |
| Δ*arlRS* | MW2 background with deletion of *arlRS* (MW1304-5) | [6] |
| Δ*arlRS* p*arlRS* | MW2 Δ*arlRS* carrying p*arlRS* plasmid | [6] |
| Δ*arlRS* P_Cd_-*mgrA* | MW2 Δ*arlRS* expressing the *mgrA* gene from the chromosome under the cadmium inducible promoter | [7] |
| Δ*mgrA* | MW2 background with deletion of *mgrA* (MW0648) | [7] |
| WT pP*_tarM_*-*gfp* | MW2 WT carrying pP*_tarM_*-*gfp* plasmid expressing sGFP under the *tarM* promoter | This study |
| Δ*arlRS* pP*_tarM_*-*gfp* | MW2 Δ*arlRS* carrying pP*_tarM_*-*gfp* plasmid expressing sGFP under the *tarM* promoter | This study |
| Δ*mgrA* pP*_tarM_*-*gfp* | MW2 Δ*mgrA* carrying pP*_tarM_*-*gfp* plasmid expressing sGFP under the *tarM* promoter | This study |
| WT pP*_mgrA_-gfp* | MW2 WT carrying pP*_mgrA_-gfp* plasmid expressing sGFP under the *mgrA* promoter | This study |
| WT pP*_spx_*-*gfp* | MW2 WT carrying pP*_spx_*-*gfp* plasmid expressing sGFP under the *spx* promoter | This study |
| Plasmids | | |
| pCN51 | Low copy number complementation vector with cadmium inducible promoter | [8] |
| p*arlRS* | pCN51 plasmid carrying *arlRS* gene | [6] |
| pCM29 | Fluorescent reporter plasmid for sGFP under *sarA*-P1 promoter | [9, 10] |
| pP*_tarM_*-*gfp* | pCM29 plasmid expressing sGFP under the *tarM* promoter | This study |
| pP*_mgrA_*-*gfp* | pCM29 plasmid expressing sGFP under the *mgrA* promoter | This study |
| pP*_spx_*-*gfp* | pCM29 plasmid expressing sGFP under the *spx* promoter | This study |
| Phages | | |
| Stab20 | Lytic myophage within the genus Kayvirus in the subfamily Twortvirinae. | [11] |
| vB_SauM_EW18 | Myophage with similar receptor binding proteins as Stab20 | [12, 13] |
| vB_SauM_EW26 | Myophage with similar receptor binding proteins as Stab20 | [12, 13] |
| vB_SauM_EW29 | Myophage with similar receptor binding proteins as Stab20 | [12, 13] |
| vB_SauM_EW72 | Myophage with similar receptor binding proteins as Stab20 | [12, 13] |

**Table S3. Overview of primers used in this study**

| Primers | RE | Sequence 5’-3’ |
| --- | --- | --- |
| Fluorescent reporter assay | | |
| Promoter *tarM* Fw | XbaI | GTA TCTAGA GTGCGGTAGAAGAATTTG |
| Promoter *tarM* Rv | KpnI | CCC GGTACC AGCGCACCTCTTAATAA |
| Promoter *mgrA* Fw | XbaI | GTA TCTAGA GTACCGAATTCATTCATGATGATTTTAATTTTC |
| Promoter *mgrA* Rv | KpnI | CCC GGTACC AATAAGAATATCCATAATTAACGGATTTTTGG |
| Promoter *spx* Fw | XbaI | GTA TCTAGA GTGTAGAAACTTATCTGAAAAATTAGGTTTTTC |
| Promoter *spx* Rv | KpnI | CCC GGTACC CTAAAATTCTTAATCGTTACTATTTATTATAACTATC |
| RT-qPCR | | |
| Amplicon *tarM* Fw | - | ATGAAAAAAATATTTATGATGGTACATG |
| Amplicon *tarM* Rv | - | GCCACTTCAATAAGCAAATC |
| Amplicon *tarS* Fw | - | TGTTCCAGGTAAAATTGTGCAATC |
| Amplicon *tarS* Rv | - | CTACACGTTCTGGAACTGCTTG |
| Amplicon *mgrA* Fw | EcoRI | GTA GAATTC ATGTCTGATCAACATAATTTAAAAGAACAGC |
| Amplicon *mgrA* Rv | BglII | CCC AGATCT TTATTTTTCCTTTGTTTCATCAAATGCATG |
| qPCR *tarM* Fw | - | GATTTGTGATGGACCAGGG |
| qPCR *tarM* Rv | - | CCGTTAATTTTATTCGCATTCTC |
| qPCR *tarS* Fw | - | GGCGTTAAAACAAGCAGAAG |
| qPCR *tarS* Rv | - | CACCACGACCATTAACACC |
| qPCR *mgrA* Fw | - | GCTCAAAGACAAGTTAATCGCTAC |
| qPCR *mgrA* Rv | - | CGACTTTCTTGACGTTTACAGG |

Underlined nucleotides indicate restriction enzyme (RE) sites. Note that RE sites of primers *Amplicon mgrA Fw* and -*Rv* were not used.

**Supplemental References**

1. Monk IR, Shah IM, Xu M, Tan MW, Foster TJ. Transforming the untransformable: application of direct transformation to manipulate genetically *Staphylococcus aureus* and *Staphylococcus epidermidis*. mBio. 2012;3(2). doi: 10.1128/mBio.00277-11.

2. Fey PD, Endres JL, Yajjala VK, Widhelm TJ, Boissy RJ, Bose JL, et al. A genetic resource for rapid and comprehensive phenotype screening of nonessential *Staphylococcus aureus* genes. mBio. 2013;4(1):e00537-12. doi: 10.1128/mBio.00537-12.

3. Diep BA, Gill SR, Chang RF, Phan TH, Chen JH, Davidson MG, et al. Complete genome sequence of USA300, an epidemic clone of community-acquired meticillin-resistant *Staphylococcus aureus*. Lancet. 2006;367(9512):731-9. doi: 10.1016/s0140-6736(06)68231-7.

4. Krusche J, Beck C, Lehmann E, Gerlach D, Wolz C, Peschel A. Systematic classification of phage receptor-binding proteins predicts surface glycopolymer structure in *Staphylococcus pathogens*. bioRxiv. 2024:2024.03.04.583386. doi: 10.1101/2024.03.04.583386.

5. Baba T, Takeuchi F, Kuroda M, Yuzawa H, Aoki K, Oguchi A, et al. Genome and virulence determinants of high virulence community-acquired MRSA. Lancet. 2002;359(9320):1819-27. doi: 10.1016/s0140-6736(02)08713-5.

6. Villanueva M, Garcia B, Valle J, Rapun B, Ruiz de Los Mozos I, Solano C, et al. Sensory deprivation in *Staphylococcus aureus*. Nat Commun. 2018;9(1):523. doi: 10.1038/s41467-018-02949-y.

7. Burgui S, Gil C, Solano C, Lasa I, Valle J. A Systematic Evaluation of the Two-Component Systems Network Reveals That ArlRS Is a Key Regulator of Catheter Colonization by *Staphylococcus aureus*. Front Microbiol. 2018;9:342. doi: 10.3389/fmicb.2018.00342.

8. Charpentier E, Anton AI, Barry P, Alfonso B, Fang Y, Novick RP. Novel cassette-based shuttle vector system for gram-positive bacteria. Appl Environ Microbiol. 2004;70(10):6076-85. doi: 10.1128/AEM.70.10.6076-6085.2004.

9. de Jong NW, van der Horst T, van Strijp JA, Nijland R. Fluorescent reporters for markerless genomic integration in *Staphylococcus aureus*. Sci Rep. 2017;7:43889. doi: 10.1038/srep43889.

10. Pang YY, Schwartz J, Thoendel M, Ackermann LW, Horswill AR, Nauseef WM. agr-Dependent interactions of *Staphylococcus aureus* USA300 with human polymorphonuclear neutrophils. J Innate Immun. 2010;2(6):546-59. doi: 10.1159/000319855.

11. Oduor JMO, Kiljunen S, Kadija E, Mureithi MW, Nyachieo A, Skurnik M. Genomic characterization of four novel Staphylococcus myoviruses. Arch Virol. 2019;164(8):2171-3. doi: 10.1007/s00705-019-04267-0.

12. Whittard E, Redfern J, Xia G, Millard A, Ragupathy R, Malic S, et al. Phenotypic and Genotypic Characterization of Novel Polyvalent Bacteriophages With Potent In Vitro Activity Against an International Collection of Genetically Diverse *Staphylococcus aureus*. Front Cell Infect Microbiol. 2021;11:698909. doi: 10.3389/fcimb.2021.698909.

13. Yang J, Bowring JZ, Krusche J, Lehmann E, Bejder BS, Silva SF, et al. Cross-species communication via *agr* controls phage susceptibility in *Staphylococcus aureus*. Cell Rep. 2023;42(9):113154. doi: 10.1016/j.celrep.2023.113154.
